# Hypoxic CA9-high mesenchymal-like glioma stem cells promote ANXA1-FPR1-dependent macrophage polarization in recurrent glioblastoma

**DOI:** 10.1186/s12974-026-03905-0

**Published:** 2026-06-29

**Authors:** Hangqi Gao, Guiting You, Jianhuang Huang, Yijing Lin, Jiexin Huang, Risheng Liang, Caihou Lin

**Affiliations:** 1 Department of Neurosurgery, Fujian Medical University Union Hospital, Fuzhou, Fujian China; 2 Department of Plastic Surgery, Fujian Medical University Union Hospital, Fuzhou, Fujian China; 3 Department of Neurosurgery, Affiliated Hospital of Putian University, Putian, Fujian China

**Keywords:** recurrent glioblastoma, carbonic anhydrase 9, annexin A1, FPR1, tumor-associated macrophages, neuroinflammation, glioma stem cells, single-cell RNA sequencing

## Abstract

**Background:**

Recurrent glioblastoma (rGBM) is characterized by marked myeloid remodeling and a profoundly immunosuppressive microenvironment. Although carbonic anhydrase 9 (CA9) is a canonical hypoxia-inducible molecule linked to aggressive glioblastoma (GBM) behavior, its cell-state-specific distribution in rGBM and its role in glioma stem cell (GSC)-macrophage crosstalk remain incompletely understood.

**Methods:**

We integrated public transcriptomic cohorts, single-cell RNA sequencing, quantitative proteomics, paired clinical specimens, and in vitro/in vivo functional assays to define the role of CA9 in rGBM. Malignant-cell states and tumor-associated macrophage (TAM) subpopulations were resolved at single-cell resolution, and intercellular communication was inferred computationally. CA9 gain- and loss-of-function models were established in GBM cell lines and patient-derived GSCs. ANXA1 expression and secretion were assessed by qRT-PCR, Western blotting, ELISA, and promoter-reporter assays. Macrophage polarization was evaluated using conditioned-medium transfer, recombinant ANXA1 rescue, and pharmacological FPR1 blockade with cyclosporin H.

**Results:**

CA9 was upregulated in GBM, further enriched in mesenchymal and recurrent tumors, and associated with inferior survival. Single-cell analysis localized CA9 predominantly to a hypoxia-associated MES-like GSC subpopulation that expanded in recurrent samples and exhibited elevated stemness. Recurrent tumors also displayed increased SPP1 + immunosuppressive TAMs. Proteomic, transcriptomic, and cell–cell communication analyses prioritized ANXA1 as a CA9-associated downstream mediator with secreted immunomodulatory potential. Hypoxia induced both CA9 and ANXA1, and mutation of a hypoxia-response element attenuated ANXA1 promoter activation. CA9 depletion reduced ANXA1 expression and secretion, impaired GBM growth phenotypes, and attenuated hypoxia-driven ANXA1 induction. CellChat highlighted enhanced signaling between hypoxic MES-like GSCs and SPP1 + TAMs in recurrent GBM, nominating ANXA1-FPR1 as a leading ligand-receptor axis. Functionally, conditioned medium from CA9-high GBM cells promoted M2-like macrophage polarization, which was weakened by CA9 knockdown, partially restored by recombinant ANXA1, and markedly suppressed by FPR1 antagonism, supporting ANXA1 as a contributory paracrine mediator, and markedly suppressed by FPR1 antagonism. In vivo, CA9 depletion, and in CA9-intact tumors FPR1 blockade, reduced M2-like macrophage-associated signals.

**Conclusions:**

CA9 is enriched in hypoxic MES-like GSCs in rGBM and promotes an ANXA1-dependent paracrine program that supports immunosuppressive macrophage polarization. These findings identify a CA9-ANXA1-FPR1-associated neuroimmune crosstalk axis linking hypoxic stem-like tumor states to myeloid remodeling in recurrent GBM, with ANXA1 functioning as a contributory paracrine mediator rather than a sole effector of CA9-driven immunosuppression.

**Graphical abstract:**

In recurrent glioblastoma, CA9 is preferentially enriched in hypoxia-associated MES-like glioma stem cells. Elevated CA9 enhances ANXA1 expression and secretion under hypoxic conditions. Secreted ANXA1 engages FPR1-associated signaling in SPP1 + tumor-associated macrophages, promoting an M2-like immunosuppressive phenotype. This tumor-to-myeloid paracrine circuit contributes to neuroimmune remodeling and supports recurrent GBM progression.

**Figure:**
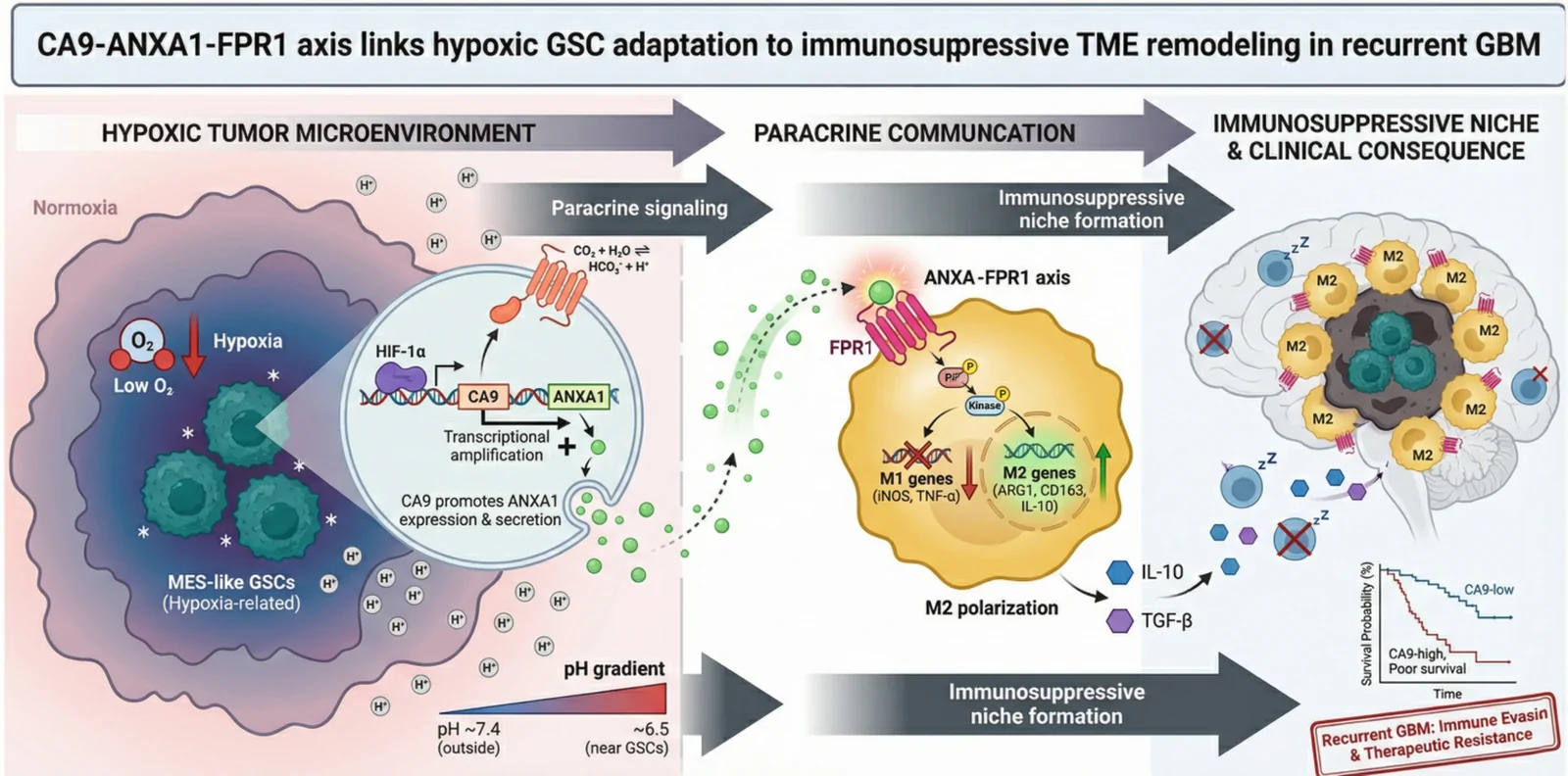

**Supplementary Information:**

The online version contains supplementary material available at https://doi.org/10.1186/s12974-026-03905-0.

## Background

Glioblastoma (GBM) is the most malignant and aggressive primary brain tumor of the central nervous system, defined by diffuse infiltration, rapid growth, limited responsiveness to radio- and chemotherapy, and near-universal recurrence [1–4]. The Stupp regimen remains the standard of care, yet median overall survival seldom exceeds 15 months from diagnosis [2, 4, 5]. Recurrence is virtually inevitable and represents the principal cause of treatment failure. Compared with primary GBM, recurrent disease (rGBM) typically harbors greater molecular heterogeneity, more deeply entrenched therapeutic resistance, and a profoundly remodeled microenvironment, making it one of the most challenging problems in neuro-oncology [3, 6–8].

A growing body of evidence positions glioma stem cells (GSCs) as the cellular foundation of GBM persistence, therapy resistance, and relapse [9–13]. Relative to bulk tumor cells, GSCs possess robust self-renewal capacity, multilineage differentiation potential, and remarkable adaptability to therapeutic pressure. Among the major glioma cell states, the mesenchymal (MES)-like state is associated with heightened invasiveness, greater treatment tolerance, and stronger immunomodulatory activity, and is widely considered central to malignant progression and recurrence [11, 14, 15]. How recurrence-associated GSC subpopulations interact with myeloid cells at single-cell resolution remains incompletely understood [10, 13].

Tumor-associated macrophages (TAMs) are among the most abundant immune populations in GBM, and macrophage-rich glioma microenvironments are increasingly recognized as neuroinflammatory ecosystems in which tumor cells, microglia, and bone marrow-derived macrophages exchange signals that promote immune evasion, angiogenesis, extracellular matrix remodeling, and therapeutic resistance [16–20]. Annexin A1 (ANXA1) is a pleiotropic immunomodulatory protein that signals through formyl peptide receptor family members to regulate inflammatory responses, cell migration, and macrophage function [21–24]. Whether ANXA1 contributes to GSC-TAM communication in recurrent GBM has not been clearly established.

Hypoxia is a hallmark of the GBM microenvironment and deeply influences both tumor-cell plasticity and immune-cell behavior [25–28]. Carbonic anhydrase 9 (CA9), a prototypical hypoxia-responsive gene regulated by HIF-1alpha, is aberrantly expressed in multiple malignancies including GBM [29–32]. By catalyzing the reversible interconversion of CO2, bicarbonate, and protons, CA9 promotes intracellular alkalinization and extracellular acidification, thereby supporting tumor-cell survival and invasion in hostile microenvironments [30, 33–36]. However, whether CA9 preferentially accumulates in recurrence-associated GSC states and whether it participates in tumor-myeloid communication in rGBM remain open questions. The rationale for focusing on CA9 was not based solely on its role as a canonical hypoxia marker. Rather, CA9 was positioned at the intersection of hypoxic adaptation, mesenchymal-like tumor-cell plasticity, recurrence-associated GSC states, and myeloid remodeling—a convergence that distinguished it from other hypoxia-responsive genes. Importantly, while hypoxia-driven ANXA1 regulation has been previously reported, the present study specifically asked whether CA9 makes a functional contribution to the amplitude of ANXA1 induction and secretion, and whether this contribution is relevant to tumor-to-macrophage paracrine communication in recurrent GBM.

Rapid advances in single-cell RNA sequencing (scRNA-seq) have greatly facilitated dissection of GBM heterogeneity at single-cell resolution [14, 15, 37, 38]. Building on this foundation, the present study integrates single-cell transcriptomics, quantitative proteomics, public cohort analyses, clinical sample validation, and in vitro/in vivo functional experiments to systematically interrogate the biological role and molecular mechanism of CA9 in rGBM. We show that CA9 is enriched in a hypoxia-associated MES-like GSC subpopulation in rGBM, promotes ANXA1 expression and secretion, and supports macrophage polarization through an ANXA1-FPR1-associated signaling axis. Collectively, these data position CA9 at the intersection of hypoxic adaptation, stem-like tumor states, and neuroimmune remodeling in recurrent GBM.

## Methods

### Clinical sample collection

**Fig. 1:**
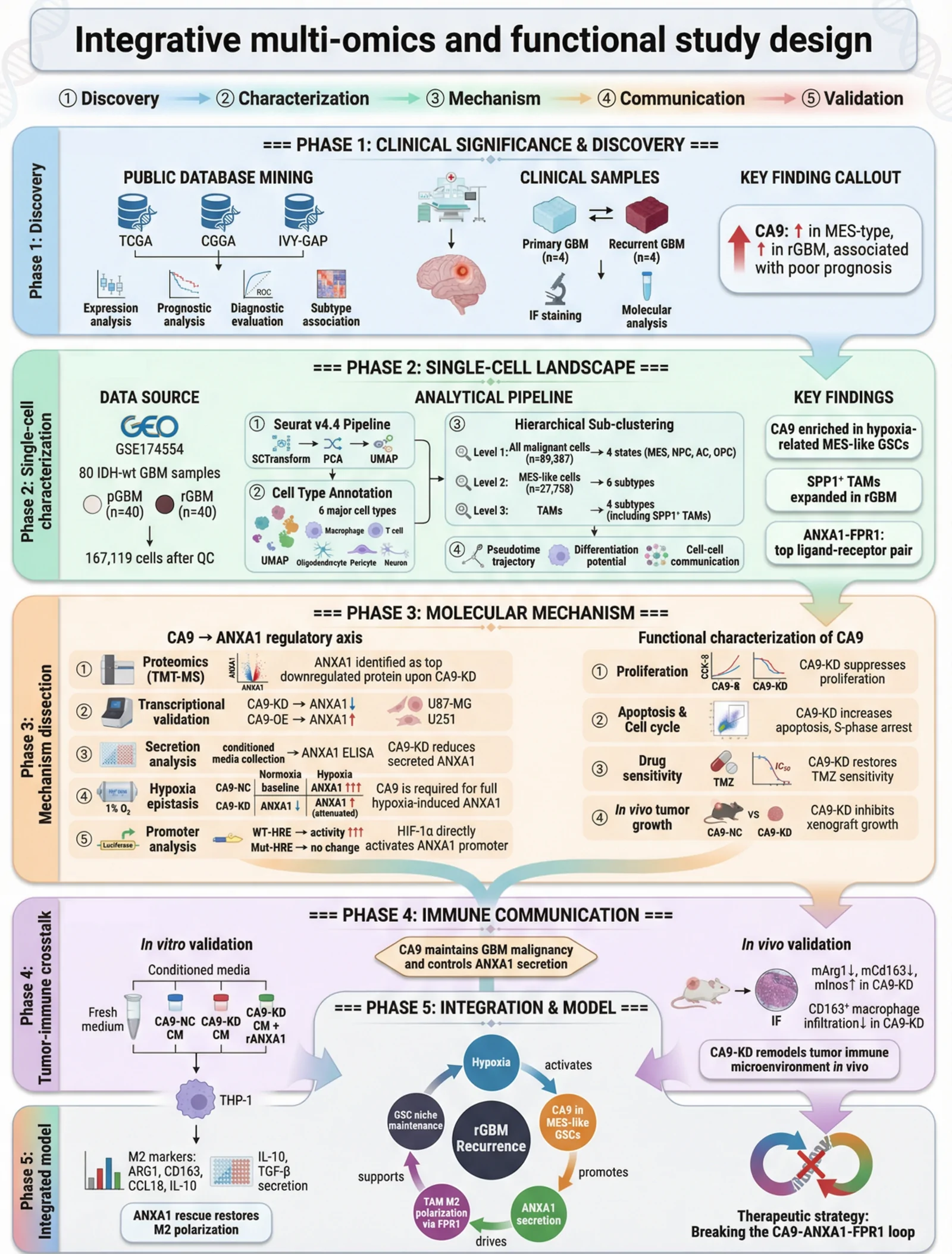
The technical route of this study

Paired primary and recurrent GBM tissue specimens were collected from patients who underwent surgical resection at the Department of Neurosurgery, Union Hospital, Fujian Medical University, between 2018 and 2023 (*n* = 4 pairs). Written informed consent was obtained from all participants. The study protocol was approved by the Institutional Review Board of Union Hospital, Fujian Medical University (approval no. 2023kjcx034) and registered with the Chinese Clinical Trial Registry (ChiCTR2200055915). Inclusion criteria: (1) histopathologically confirmed IDH-wild-type GBM per the 2021 WHO CNS tumor classification [1]; (2) complete clinical data and follow-up records; (3) primary specimens collected at initial surgery before any radio- or chemotherapy; and (4) recurrent specimens obtained at reoperation after standard treatment, with recurrence confirmed by MRI and/or histopathology. Exclusion criteria: concurrent malignancy, tissue quality insufficient for downstream analysis, or incomplete clinical records. Fresh tumor tissue was processed within 30 min of resection: a portion was snap-frozen in liquid nitrogen for molecular analysis and the remainder fixed in 4% paraformaldehyde for histopathology. The overall study workflow is illustrated in Fig. 1.

### Cell culture

Human glioma cell lines U87-MG and U251 were obtained from the Cell Bank of the Chinese Academy of Sciences (Shanghai) and maintained in high-glucose DMEM (Yuanpei, L110KJ) supplemented with 10% fetal bovine serum (Tianhang, 11011 − 8611) and 1% penicillin–streptomycin (Beyotime, C0222) at 37 °C in 5% CO₂. THP-1 human monocytic cells were cultured in RPMI-1640 (Yuanpei, L210KJ) with 10% FBS. All lines were authenticated by STR profiling and routinely screened for mycoplasma. Patient-derived GSCs were isolated from fresh GBM tissue and propagated in serum-free DMEM/F12 supplemented with B27, EGF, and bFGF under non-adherent sphere-forming conditions; identity was confirmed by immunostaining for Nestin and SOX2 [9, 12].

### Lentiviral transduction and stable cell line generation

CA9-targeting shRNA lentiviruses, CA9-overexpression lentiviruses, and matched controls were purchased from GeneChem (Shanghai). The CA9 shRNA sequence was 5’-TTGGAAGAAATCGCTGAGGAA-3’; the non-targeting control was 5’-TTCTCCGAACGTGTCACGT-3’. Cells at 40–50% confluence were transduced at MOI = 20 with polybrene (Beyotime, C0351; 5 µg/mL). Medium was replaced at 12 h; puromycin selection (Beyotime, ST551; 2 µg/mL) began at 48 h post-transduction. Stable lines were established after two weeks of selection and confirmed by qRT-PCR and Western blotting. Key conclusions were cross-validated at mRNA and protein levels to minimize off-target bias.

### Hypoxia treatment

Hypoxia was generated in a tri-gas incubator set to 1% O₂, 5% CO₂, and 94% N₂ for 48 h. Normoxic controls were maintained at 21% O₂ with 5% CO₂. To minimize potential reoxygenation artifacts, cells and conditioned medium were collected immediately upon completion of the hypoxic period. All samples were lysed on ice and handled at low temperature throughout processing. This 1% O₂ for 48 h condition follows established protocols for modeling GBM tumor hypoxia [25, 29, 32].

### Conditioned medium preparation and macrophage polarization assay

THP-1 cells were differentiated with 100 ng/mL PMA (Beyotime, S1819) for 24 h, washed three times with PBS, and rested in fresh medium for a further 24 h. Conditioned medium (CM) from CA9-NC or CA9-KD glioma cells cultured for 48 h was filtered through a 0.22-micrometer membrane and diluted 1:1 with fresh RPMI-1640 before addition to macrophage cultures. CM was collected from cultures initiated at identical seeding densities to minimize cell-number-dependent variation in secreted protein concentrations. For ANXA1 rescue experiments, recombinant human ANXA1 (Cloud-Clone Corp., RPA511Hu01; 100 ng/mL) was supplemented to CA9-KD CM. After 48 h, cells were harvested for qRT-PCR and supernatants collected for ELISA.

### FPR1 inhibition experiments

THP-1-derived macrophages were pretreated with the selective FPR1 antagonist cyclosporin H (CsH, 10 micromolar) or vehicle control for 1 h before conditioned-medium stimulation. For immediate-early response analyses, macrophages were harvested after 1 h and analyzed for EGR1, FOS, and CXCL8 expression. For polarization assays and cytokine measurements, cells and supernatants were collected after 48 h for qRT-PCR and ELISA. For in vivo experiments, mice bearing CA9-NC or CA9-KD subcutaneous xenografts were randomized into four groups.

(*n* = 8 per group): CA9-NC + vehicle, CA9-NC + CsH, CA9-KD + vehicle, and CA9-KD + CsH. CsH (10 mg/kg; MedChemExpress, HY-100183) dissolved in vehicle (10% DMSO, 40% PEG300, 5% Tween 80, 45% saline) or vehicle alone was administered intraperitoneally every other day beginning on day 3 after implantation. Mice were sacrificed on day 21, and tumors.

were processed as described above.

### RNA extraction and quantitative real-time PCR

Total RNA was extracted with TRIzol (Tiangen, DP424). RNA purity was assessed by A260/A280 ratio (acceptable range 1.8-2.0). One microgram of total RNA was reverse-transcribed using PrimeScript RT Master Mix (Takara, RR036A; 37 degrees C for 15 min, then 85 degrees C for 5 s). Quantitative PCR was run with TB Green Premix Ex Taq II (Takara, RR820A) in 20-microliter reactions. Expression was normalized to beta-actin using the 2^-delta delta Ct method. For human glioma cells and THP-1-derived macrophages, primers included CA9, ANXA1, HIF1A, ARG1, CD163, CCL18, IL10, NOS2, TNFA, IL1B, EGR1, FOS, and CXCL8. For xenograft tissue, mouse-specific primers for Arg1, Cd163, and Nos2 were used. Primer sequences are provided in Supplementary Table 1.

### Protein extraction and Western blotting

Cells were lysed in RIPA buffer (Beyotime, P0013B) supplemented with protease and phosphatase inhibitors. Lysates were cleared at 12,000 × g for 15 min at 4 °C; protein concentrations were determined by BCA assay (Beyotime, P0012). Thirty micrograms of protein was resolved by 10% SDS-PAGE, transferred to PVDF membranes, blocked with 5% non-fat milk for 1 h, and incubated overnight at 4 °C with primary antibodies: CA9 (Proteintech, 66243-1-Ig; 1:2000), ANXA1 (Proteintech, 21990-1-AP; 1:1000), HIF-1α (Proteintech, 20960-1-AP; 1:1000), and β-actin (Proteintech, 66009-1-Ig; 1:5000). HRP-conjugated secondary antibodies (Proteintech, SA00001-2 or SA00001-1; 1:5000) were applied for 1 h at room temperature. Signals were detected by ECL (Beyotime, P0018S) and quantified with ImageJ.

### Enzyme-linked immunosorbent assay

Culture supernatants were cleared by centrifugation at 1,000 x g for 10 min at 4 degrees C. Concentrations of ANXA1 (Elabscience, E-EL-H0484c), IL-10 (Elabscience, E-EL-H0103c), TGF-beta1 (Elabscience, E-EL-H0110c), and, where indicated, CCL18 were quantified by ELISA according to the manufacturers’ instructions.

### Cell proliferation and TMZ sensitivity assays

Cells were seeded at 2,000 cells/well in 96-well plates (6 replicates per group). CCK-8 reagent (Beyotime, C0038; 10 µL/well) was added at 0, 24, 48, 72, and 96 h; absorbance was read at 450 nm after 2 h at 37 °C. For TMZ sensitivity, cells received graded TMZ concentrations (0, 31.25, 62.5, 125, 250, 500, and 1000 µM; MedChemExpress, MB1160) for 72 h, and CCK-8 was performed thereafter. Viability (%) = [(experimental OD − blank OD) / (control OD − blank OD)] × 100. IC50 values were calculated in GraphPad Prism 9.0. The TMZ concentration range follows published protocols for GBM drug-resistance studies [5, 12].

### Flow cytometry

For apoptosis, ~ 1 × 10⁶ cells were stained with the Annexin V-FITC/PI Apoptosis Detection Kit (KeyGen, KGA108) for 15 min at room temperature in the dark and immediately analyzed. For cell cycle, cells were fixed overnight in 70% ethanol and processed with the Cell Cycle Analysis Kit (Beyotime, C1052). Data were acquired on a flow cytometer and analyzed with FlowJo V10.

### Immunofluorescence staining

Paraffin sections were deparaffinized, rehydrated, and subjected to heat-induced antigen retrieval in citrate buffer (Beyotime, P0081; pH 6.0). Following quenching with 3% H₂O₂ and blocking with goat serum (Beyotime, C0265), sections were incubated overnight at 4 °C with primary antibodies: CA9 (Proteintech, 66243-1-Ig; 1:200), Nestin (Proteintech, 19483-1-AP; 1:100), or CD163 (Proteintech, 16646-1-AP; 1:100). Fluorescence-labeled secondary antibodies (Proteintech, SA00013-2 or SA00013-3; 1:200) were applied for 1 h at room temperature in the dark. Nuclei were counterstained with DAPI (Beyotime, C1005). Sections were mounted with anti-fade medium (Beyotime, P0126) and imaged by confocal laser-scanning microscopy. Fluorescence intensities were quantified with ImageJ.

### Dual-luciferase reporter assay

A 2200-bp ANXA1 promoter fragment (2000 bp upstream to 200 bp downstream of the transcription start site) was cloned into pGL3-Basic (Promega, Beijing). HRE mutagenesis was performed using the Vazyme mutagenesis kit (C214-01), replacing the core HRE sequence 5’-ACGTG-3’ with 5’-AAAAG-3’ to abolish HIF-1α-responsive element function. HEK293T cells were co-transfected with wild-type or HRE-mutant reporter (500 ng/well) and pRL-TK (50 ng/well) using Lipofectamine (Beyotime, C0526). At 24 h post-transfection, cells were transferred to normoxic or hypoxic conditions for a further 24 h. Firefly and Renilla luciferase activities were measured sequentially with the Dual-Luciferase Reporter Assay System (Beyotime, RG027), and firefly values were normalized to Renilla. The experimental design follows established HIF-1α/HRE reporter paradigms [27, 29].

### Animal experiments

All procedures complied with the Guide for the Care and Use of Laboratory Animals and adhered to the 3Rs principles (approval no. IACUC FJMU 2022 − 0559). Four-week-old female BALB/c nude mice (Slaccas, Shanghai) were housed under SPF conditions (22 ± 2 °C; 50 ± 10% humidity; 12-h light/dark cycle) with ad libitum access to food and water, and acclimatized for one week before experiments. Animals were randomized into CA9-NC and CA9-KD groups (*n* = 8 per group). Exponentially growing cells resuspended at 2.5 × 10⁷/mL were injected subcutaneously into the right flank (200 µL; 5 × 10⁶ cells). Tumor dimensions were recorded every three days; volume was calculated as V = L × W²/2. Mice were sacrificed on day 23 post-inoculation. Tumors were excised, weighed, photographed, and partitioned for molecular analysis (snap-frozen) or histology (4% paraformaldehyde). Tumor measurements and histological analyses were performed by personnel blinded to group allocation. For FPR1 inhibition experiments, a separate cohort of mice was used. Animals were randomized into four groups (CA9-NC + vehicle, CA9-NC + CsH, CA9-KD + vehicle, CA9-KD + CsH; *n* = 8 per group), and CsH was administered as described in the FPR1 inhibition experiments section. Mice were sacrificed on day 21 post-inoculation.

### Macrophage phenotype analysis in tumor tissue

Total RNA from snap-frozen xenograft tissue was subjected to qRT-PCR using mouse-specific primers for Arg1 and Cd163 (M2-like markers) and Nos2 (M1-like marker). The species-specific design exploits the xenograft architecture of human tumor cells and murine host immune infiltrates to selectively interrogate infiltrating macrophage-associated signals [16, 18].

### Quantitative proteomics

Three independent biological replicates of CA9-NC and CA9-KD U87-MG cells were processed by filter-aided sample preparation (FASP) and digested overnight with trypsin (50:1, protein: enzyme) at 37 °C. Peptides were labeled with TMT 6plex (Thermo Fisher Scientific), fractionated by high-pH reversed-phase HPLC, and analyzed by nanoLC-MS/MS on a timsTOF Pro mass spectrometer (Bruker). Database searches were performed in MaxQuant (v2.0.3.0) against the UniProt human proteome with the following settings: fixed modification, cysteine carbamidomethylation; variable modifications, methionine oxidation and N-terminal acetylation; peptide and protein FDR both < 1%. Differentially expressed proteins were defined by |log₂FC| > 1 and *P* < 0.05. GO and KEGG enrichment analyses used the DAVID database (FDR < 0.05).

To prioritize downstream mediators relevant to GSC-to-macrophage paracrine communication, differentially expressed proteins were first filtered using |log₂FC| > 1 and *P* < 0.05. We then focused on proteins downregulated after CA9 depletion with known or predicted extracellular localization, secretory potential, or immune-regulatory relevance. Remaining candidates were cross-referenced against (i) positive correlation with CA9 in public GBM cohorts, and (ii) association with macrophage abundance scores. Independent convergent support from single-cell ligand–receptor communication analysis is described in the corresponding Results section.

### Public database analyses

TCGA-GBM and TCGA-LGG expression data and clinical information were downloaded from UCSC Xena (https://xenabrowser.net/). Cases were reclassified as GBM using IDH mutation status, 1p/19q co-deletion, and TERT promoter mutation per the 2021 WHO CNS5 criteria [1]. The CGGA mRNAseq_693 dataset (http://www.cgga.org.cn/) served as an independent validation cohort. Kaplan–Meier survival analysis used the R packages survival and survminer, with log-rank testing. ROC analysis used the pROC package. Immune cell infiltration scores were calculated by ssGSEA (GSVA package), and Spearman correlation assessed associations between CA9 expression and immune cell abundance.

### Single-cell RNA sequencing data analysis

scRNA-seq data from GSE174554 (GEO; PMID: 36539501) [38], comprising 40 primary and 40 recurrent IDH-wild-type GBM samples, were processed in Seurat (v4.4.0) [39]. Quality control: 200–6,000 genes per cell; < 20% mitochondrial reads. Normalization and batch correction used SCTransform [40]. PCA (50 components) was followed by UMAP visualization using the top 20 PCs. Clusters were identified with FindNeighbors and FindClusters (resolution = 0.4). Cell-type annotation combined SingleR (Human Primary Cell Atlas) [41] with manual curation. Malignant cells were identified by inferCNV [42] using T cells as reference. Malignant cells and TAMs were each re-clustered independently; subpopulations were annotated using marker genes from published literature [14, 15, 18, 38]. Pseudotime analysis used Monocle2 (v2.26.0) with DDRTree [43]. Differentiation potential was quantified by CytoTRACE2 [44]. Cell–cell communication was inferred by CellChat (v1.6.1) [45], analyzed separately for primary and recurrent samples.

### Statistical analysis

All experiments were performed in at least three independent biological replicates. Data are presented as mean +/- SEM. Statistical analyses were conducted in R (v4.4.0). Two-group comparisons used two-tailed Student’s t-test; multi-group comparisons used one-way ANOVA with Tukey’s post-hoc test. Survival analysis used the Kaplan-Meier method with log-rank testing. Correlations were assessed by Spearman’s rank coefficient. *P* < 0.05 was considered statistically significant (*P < 0.05; **P < 0.01; ***P < 0.001; ****P < 0.0001; ns, not significant).

## Results

### CA9 is upregulated in GBM and associates with the mesenchymal subtype, recurrent disease, and poor prognosis

**Fig. 2:**
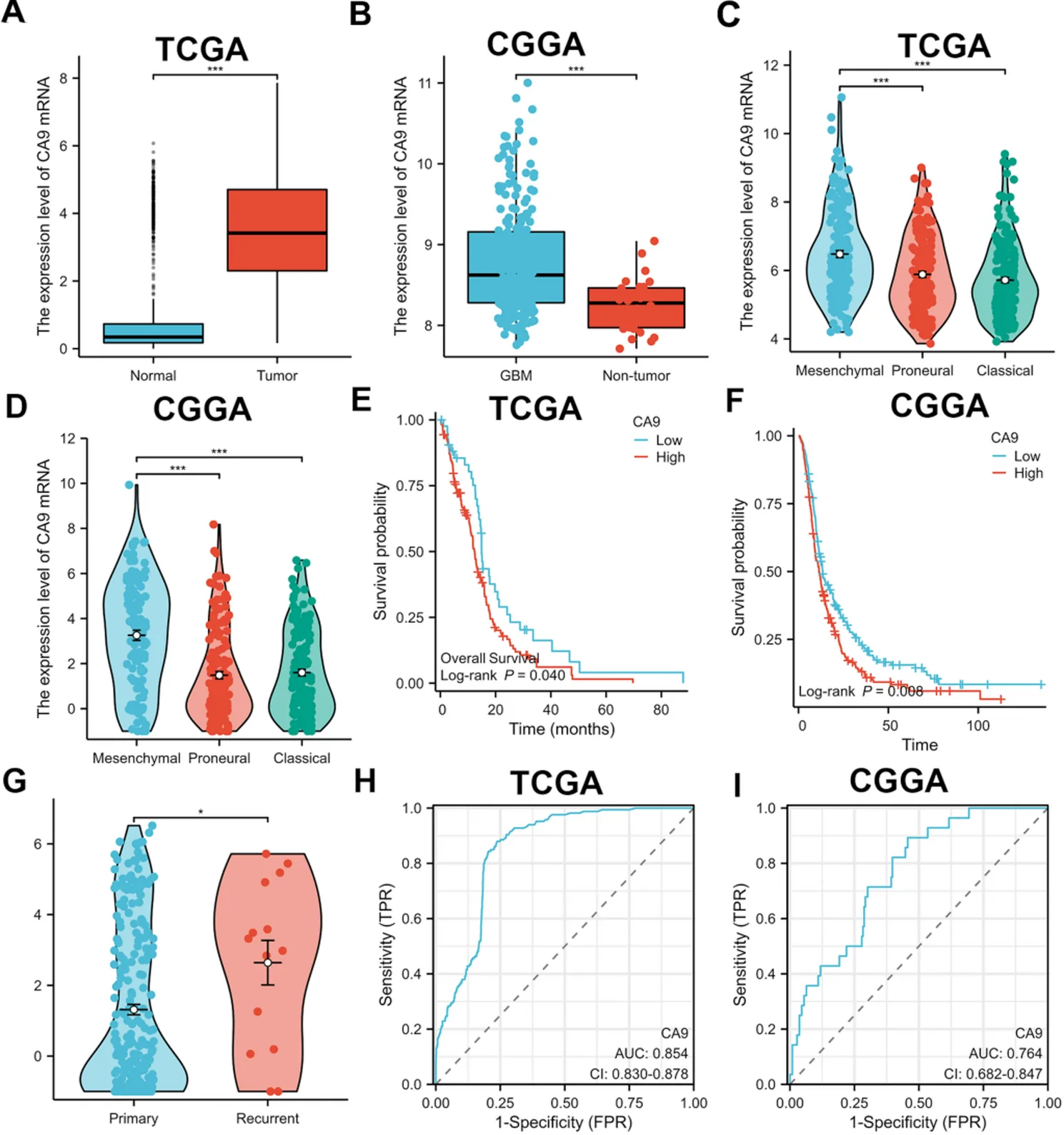
CA9 is upregulated in GBM and correlates with the mesenchymal subtype, recurrent disease, and poor prognosis. **A**, **B** CA9 expression in GBM in the TCGA and CGGA cohorts. **C**, **D** CA9 expression across GBM molecular subtypes; note significant elevation in the mesenchymal (MES) subtype. **E**, **F** Kaplan–Meier overall survival curves stratified by CA9 expression level. **G** CA9 expression comparison between primary and recurrent GBM specimens. **H**, **I** ROC curves assessing the diagnostic accuracy of CA9 for distinguishing GBM from non-tumor controls. Data derived from public database analyses. Boxplot centerlines, medians; boxes, IQR; whiskers, 1.5 × IQR. Survival differences by log-rank test; other comparisons by two-sided Wilcoxon rank-sum or Student’s t-test. *P < 0.05, ***P < 0.001

To establish the clinical relevance of CA9 in GBM, we first examined its expression across public cohorts. CA9 was significantly upregulated in IDH-wild-type GBM in both the re-classified TCGA-GBM and CGGA-GBM datasets (Fig. 2A–B). Subtype stratification revealed markedly higher CA9 expression in mesenchymal-subtype GBM relative to proneural or classical subtypes (Fig. 2C–D). Kaplan–Meier analysis demonstrated that elevated CA9 correlated with significantly shorter overall survival in both cohorts (Fig. 2E–F). Strikingly, CA9 was further increased in recurrent relative to primary GBM tissue (Fig. 2G). ROC curve analysis indicated good diagnostic performance for distinguishing GBM from controls (AUC > 0.75; Fig. 2H–I). Together, these findings link CA9 expression to GBM malignancy grade, mesenchymal identity, and recurrence-related clinical characteristics.

### CA9 knockdown suppresses GBM malignant phenotypes and sensitizes cells to temozolomide

**Fig. 3:**
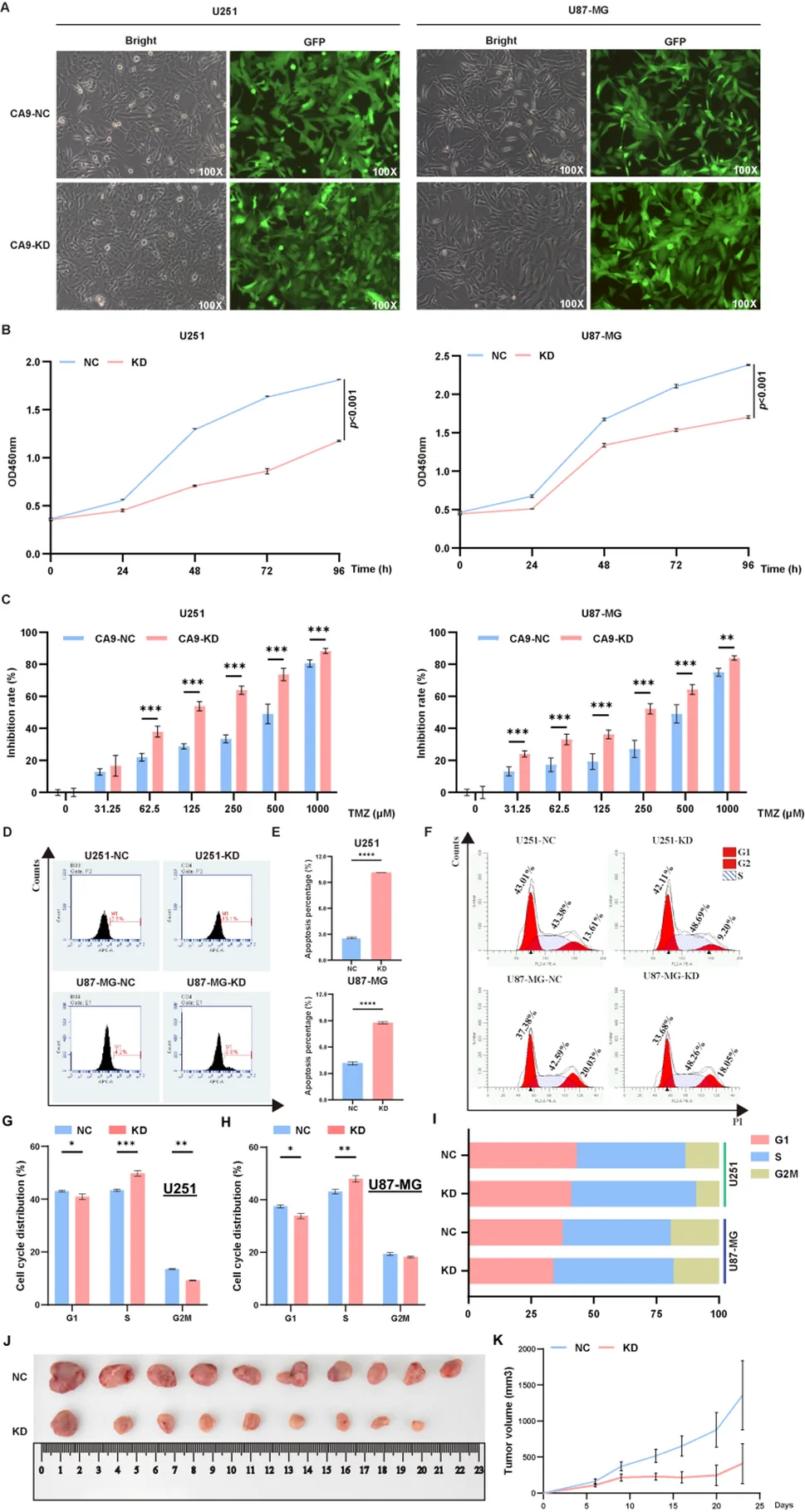
CA9 knockdown suppresses GBM malignant phenotypes and sensitizes cells to temozolomide. **A** Fluorescence micrographs illustrating lentiviral transduction efficiency in U251 and U87-MG cells. **B** CCK-8 proliferation assay after CA9 knockdown. **C** TMZ dose–response curves (72-h treatment) for IC50 estimation. **D** Representative Annexin V-FITC/PI scatter plots. **E** Quantification of apoptotic fractions. **F**–**I** Cell cycle distribution demonstrating S-phase accumulation following CA9 knockdown. **J** Tumor growth curves and endpoint photographs from the subcutaneous xenograft model. **K** Endpoint tumor weights. In vitro: ≥ 3 independent replicates. In vivo: *n* = 8 per group. Data, mean ± SEM. Two-group comparisons, two-sided Student’s t-test; tumor volume time-course, repeated-measures ANOVA. *P < 0.05, **P < 0.01, ***P < 0.001

To determine whether CA9 functionally contributes to GBM malignancy, stable CA9-knockdown (CA9-KD) lines were generated in U251 and U87-MG by lentiviral transduction (Fig. 3A). qRT-PCR confirmed CA9 mRNA reductions of 80.19% in U251 and 77.38% in U87-MG relative to non-targeting controls (Fig. S1A).

CA9 knockdown significantly impaired proliferation in both cell lines (Fig. 3B) and substantially sensitized them to TMZ (Fig. 3C). Annexin V-FITC/PI staining showed that apoptosis increased from 2.85% to 10.14% in U251 and from 4.16% to 8.76% in U87-MG following CA9 depletion (Fig. 3D–E). Cell cycle analysis revealed S-phase accumulation with reductions in G1 and G2/M fractions, indicative of S-phase arrest (Fig. 3F–I). In vivo, subcutaneous xenografts derived from CA9-KD U87-MG cells grew significantly more slowly and reached markedly lower tumor volumes and weights than controls (Fig. 3J–K; Fig. S1B). These results establish that CA9 actively sustains the proliferative and chemotherapy-tolerant phenotype of GBM cells.

### Single-cell atlas of primary and recurrent GBM reveals extensive cellular recomposition

**Fig. 4:**
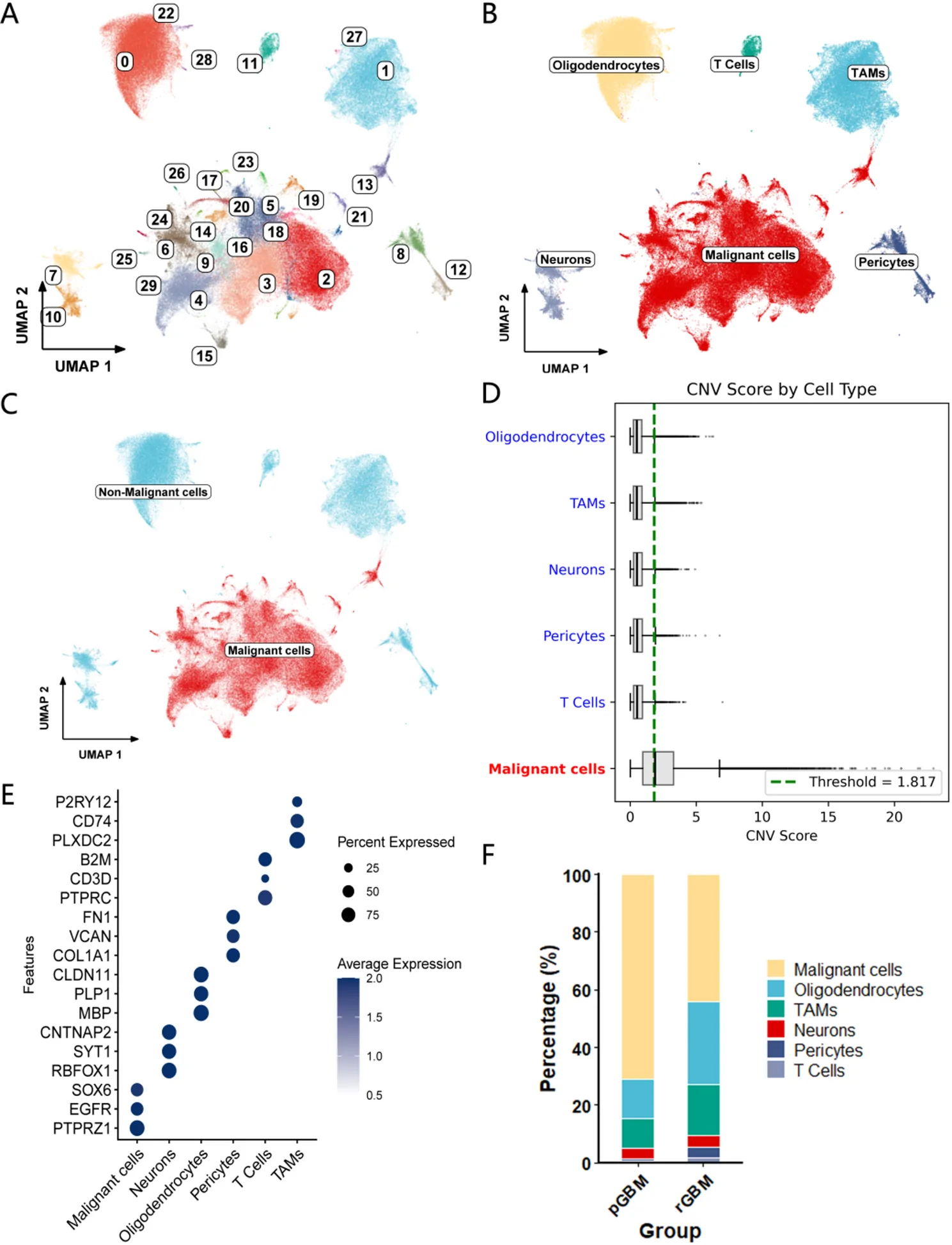
Single-cell atlas of primary and recurrent GBM reveals extensive cellular recomposition. **A** UMAP of all quality-controlled cells from 80 IDH-wild-type GBM samples, showing 30 clusters. **B** Major cell-type annotation combining marker gene expression and SingleR, comprising malignant tumor cells, macrophages, T cells, oligodendrocytes, pericytes, and neurons. **C**, **D **InferCNV copy-number variation profiles delineating malignant from non-malignant cells. **E** Marker gene expression heatmap validating cell-type annotations. **F** Relative cell-type proportions in primary versus recurrent GBM. Color intensity reflects normalized expression or relative enrichment

To systematically resolve cellular heterogeneity during GBM recurrence, we analyzed scRNA-seq data from 80 IDH-wild-type GBM samples (40 primary, 40 recurrent; GSE174554). Following quality control, 167,119 cells were retained and clustered into 30 populations (Clusters 0–29; Fig. 4A). Integration of marker gene expression with SingleR annotation defined six major cell types: malignant tumor cells, macrophages, T cells, oligodendrocytes, pericytes, and neurons (Fig. 4B). InferCNV analysis using T cells as reference corroborated the delineation of malignant from non-malignant cells (Fig. 4C–D); canonical marker expression validated all annotations (Fig. 4E). Notably, recurrent GBM exhibited a higher relative abundance of macrophages and oligodendrocytes alongside a lower proportion of tumor cells (Fig. 4F), indicating that recurrence is accompanied not only by tumor cell reprogramming but also by substantial remodeling of the immune compartment.

### CA9 is preferentially enriched in a hypoxia-associated MES-like GSC subpopulation that expands in recurrent GBM

**Fig. 5:**
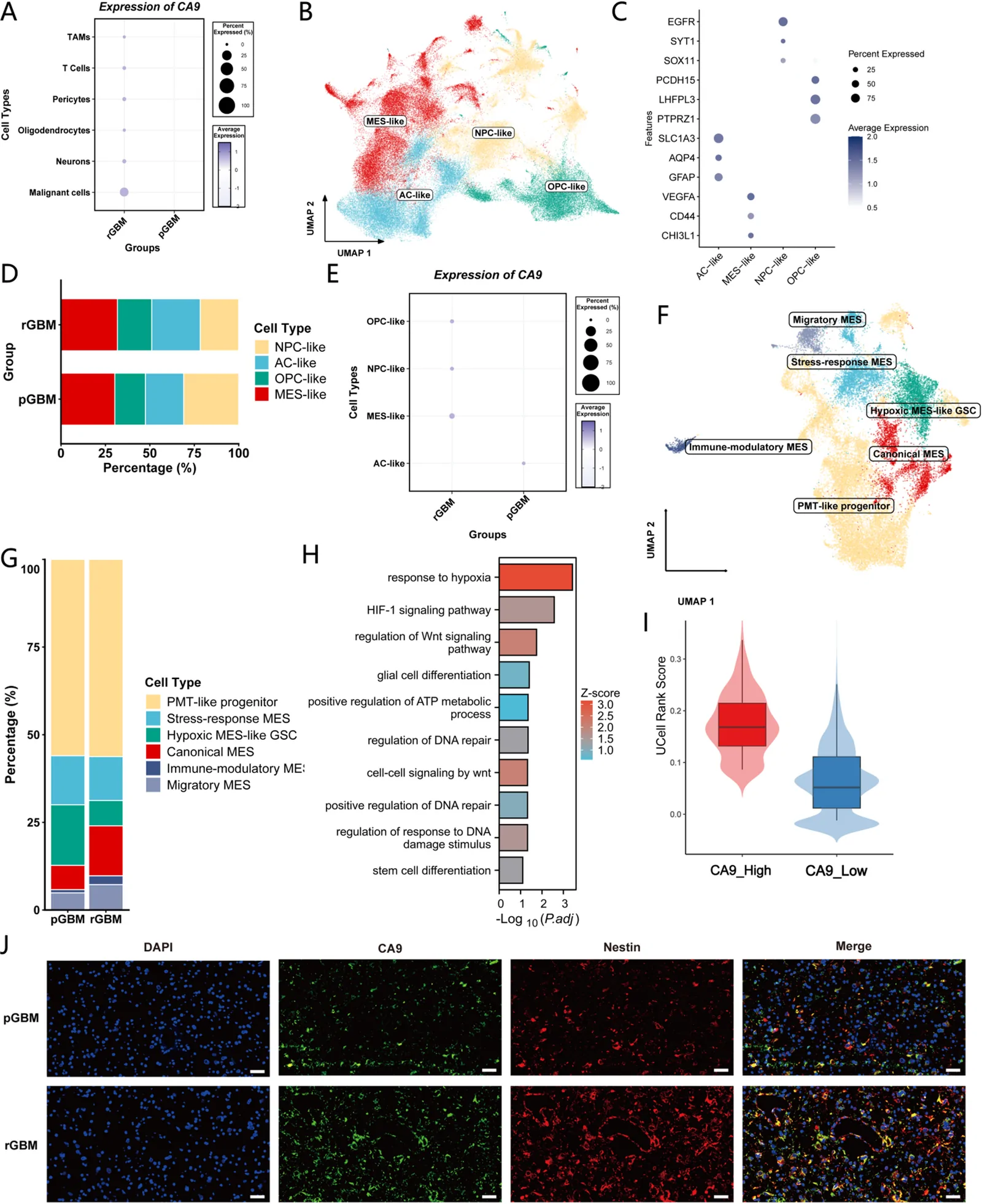
CA9 is preferentially enriched in a hypoxia-associated MES-like GSC subpopulation that expands in recurrent GBM. **A** CA9 expression comparison between primary and recurrent GBM and its distribution across major cell types. **B** UMAP of re-clustered malignant cells, delineating MES-like, NPC-like, AC-like, and OPC-like states. **C** Marker gene expression supporting cellular state annotations. **D** Proportional composition of malignant cell states in primary versus recurrent GBM. **E** CA9 expression across malignant cellular states (MES-like, NPC-like, AC-like, OPC-like), showing peak enrichment in the MES-like state. **F** UMAP of MES-like cells after secondary clustering, defining six functional substates. **G** Proportional comparison of MES-like substates, showing expansion of the hypoxia-associated MES-like GSC substate in recurrent samples. **H** GSEA enrichment analysis for the hypoxia-associated MES-like GSC substate. **I** Stemness score comparison between CA9-high and CA9-low cells within the hypoxia-associated MES-like GSC substate. **J** Immunofluorescence co-staining for CA9 and Nestin in paired primary/recurrent GBM tissue; right panels show fluorescence intensity quantification. Clinical specimens: *n* = 4 paired samples

Overall, CA9 expression was significantly higher in recurrent than in primary GBM (*P* = 6.19 × 10⁻²⁵) and was predominantly confined to the malignant cell compartment (Fig. 5A). To dissect malignant cell heterogeneity, the 89,387 malignant cells were re-clustered into 30 subpopulations (Fig. S2A) and consolidated into four major cellular states: MES-like, NPC-like, AC-like, and OPC-like (Fig. 5B–C). In recurrent GBM, MES-like and AC-like fractions trended upward while NPC-like cells declined (Fig. 5D). Among the four major malignant cellular states, CA9 expression was most prominently enriched in the MES-like state (Fig. 5E).

To characterize MES-like cell heterogeneity further, the 27,758 MES-like tumor cells were sub-clustered into 13 groups (Fig. S2B) and annotated into six functional substates (Fig. 5F): immune-modulatory MES, canonical MES, PMT-like progenitor, hypoxia-associated MES-like GSC, stress-response MES, and migratory MES. These substates reflect distinct functional programs within the MES lineage rather than fixed, discrete entities. Proportional analysis revealed that the hypoxia-associated MES-like GSC substate expanded markedly in recurrent GBM (Fig. 5G). GSEA confirmed significant enrichment for hypoxia response, DNA repair, HIF-1 signaling, and Wnt signaling pathways (Fig. 5H), consistent with a hybrid identity integrating hypoxic adaptation, damage tolerance, and stemness maintenance. Within this substate, CA9-high cells displayed higher GSC stemness scores than CA9-low cells (Fig. 5I), reinforcing the link between CA9 and the hypoxia-associated MES-like stem state in rGBM.

These computational findings were independently corroborated by immunofluorescence staining of paired clinical specimens: CA9 expression was significantly higher in recurrent versus primary GBM tissue, and Nestin enrichment was more pronounced in perivascular regions of recurrent samples (Fig. 5J). Taken together, these data suggest that CA9-high cells in rGBM define a MES-like GSC population with enhanced stemness and hypoxic adaptability.

### Pseudotime analysis and proteomics prioritize ANXA1 as a CA9-associated downstream mediator

**Fig. 6:**
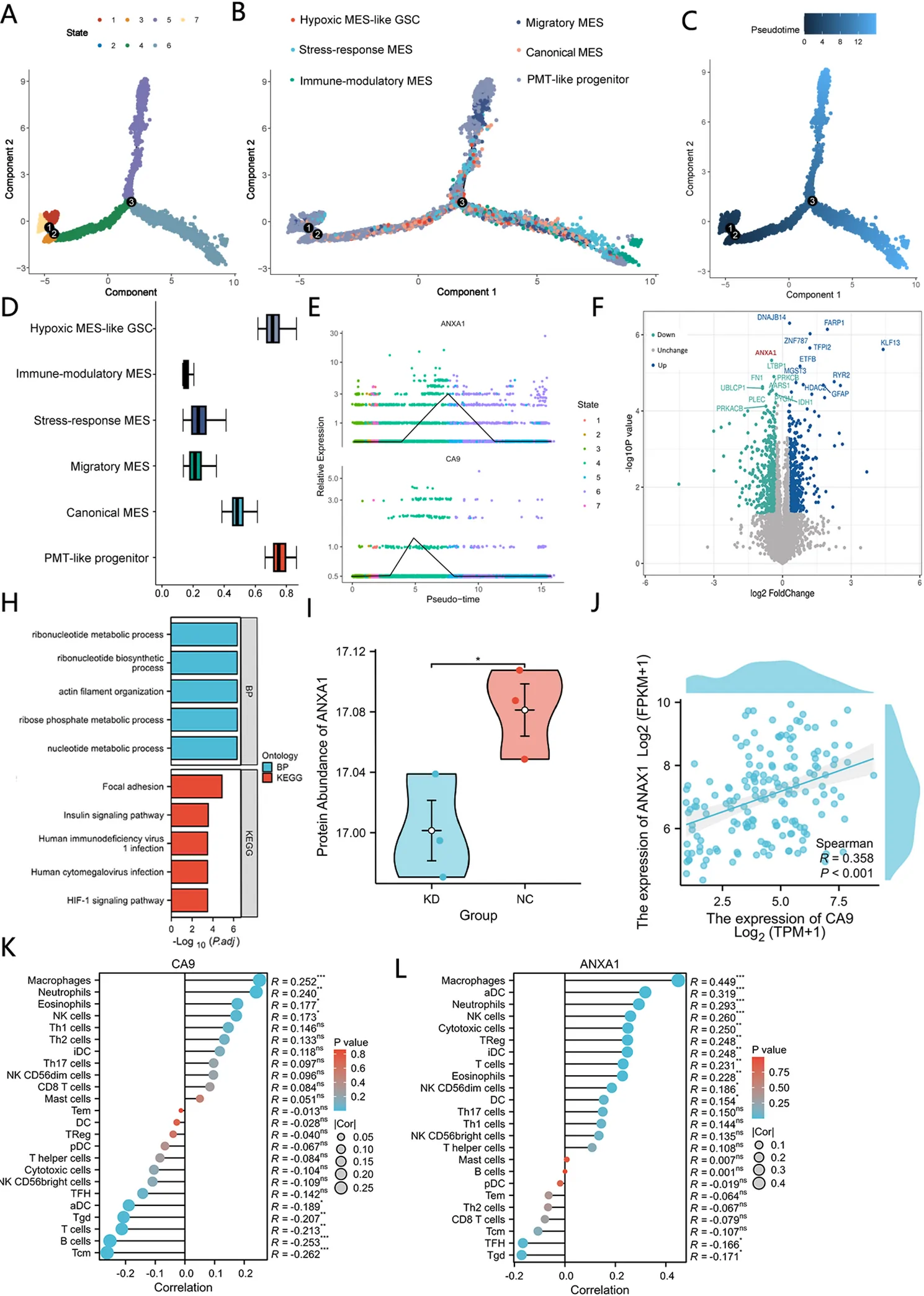
Pseudotime analysis and proteomics prioritize ANXA1 as a CA9-associated downstream mediator. **A**–**C** Pseudotime trajectory of GSC-related subpopulations in recurrent GBM, showing progression from PMT-like progenitors toward terminal states. **D** CytoTRACE2 differentiation potential scores, consistent with the pseudotime trajectory. **E** Dynamic CA9 expression along the pseudotime axis, peaking at the intermediate stage. **F** Volcano plot of differentially expressed proteins between CA9-NC and CA9-KD cells. **G** Hierarchical clustering heatmap. **H** KEGG pathway enrichment of downregulated proteins. **I** ANXA1 among differentially expressed proteins. **J** Spearman correlation between CA9 and ANXA1 mRNA in TCGA-GBM. **K**, **L** Spearman correlations between macrophage abundance and CA9 or ANXA1 expression. Proteomics: *n* = 3 biological replicates per group. *P < 0.05, **P < 0.01, ***P < 0.001

To explore developmental relationships among GSC substates, unsupervised pseudotime analysis was performed on recurrent GBM GSC-related subpopulations (Fig. 6A–C). MES-like tumor cells traversed a continuous trajectory from PMT-like progenitor origin toward terminal states. CytoTRACE2 analysis corroborated this ordering by independently capturing the differentiation potential gradient (Fig. 6D). CA9 expression peaked at an intermediate transitional stage along the pseudotime axis—corresponding predominantly to the hypoxia-associated MES-like GSC substate—before declining as differentiation progressed (Fig. 6E), suggesting that CA9 may facilitate the acquisition of more adaptive, malignant cellular states.

To identify downstream effectors, we performed quantitative proteomics on CA9-NC and CA9-KD U87-MG cells. Differential analysis yielded 707 upregulated and 685 downregulated proteins (Fig. 6F–G). KEGG enrichment of downregulated proteins highlighted focal adhesion, insulin signaling, and HIF-1 signaling pathways (Fig. 6H). We applied a biologically informed, multi-layered prioritization strategy to the CA9-regulated proteomic candidates. Given our research focus on GSC-to-macrophage paracrine communication, we prioritized proteins reduced after CA9 knockdown that exhibited known or predicted secretory, extracellular, or immunomodulatory potential. Among these candidates, ANXA1 was notable for several converging reasons: it was consistently downregulated following CA9 depletion across replicates (Fig. 6I); it has established relevance to inflammatory resolution, immune cell migration, and formyl peptide receptor family signaling; its mRNA expression positively correlated with CA9 in TCGA-GBM (Fig. 6J); and macrophage abundance scores positively correlated with both CA9 (*R* = 0.252) and ANXA1 (*R* = 0.449) expression in public GBM cohorts (Fig. 6K-L). Together, these criteria nominated ANXA1 as a priority candidate mediator linking CA9 activity to macrophage-related microenvironmental remodeling, warranting functional validation.

### CA9 positively regulates ANXA1 at both transcriptional and secretory levels

**Fig. 7:**
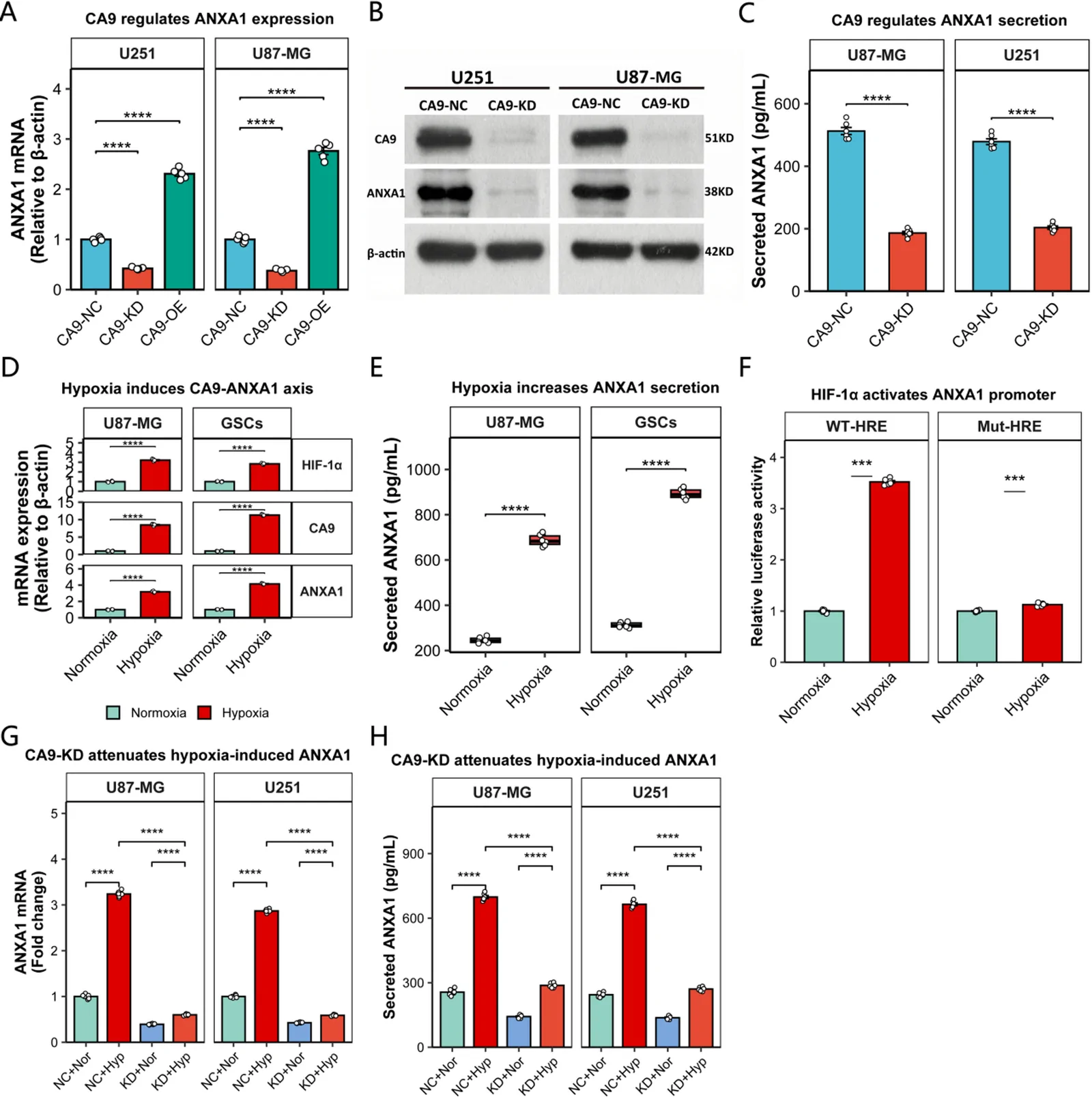
CA9 positively regulates ANXA1 at transcriptional and secretory levels and is required for full ANXA1 induction under hypoxia. All hypoxia experiments were performed at 1% O₂ for 48 h unless otherwise stated. Samples were collected immediately upon hypoxic treatment completion. **A** qRT-PCR of ANXA1 mRNA following CA9 knockdown or overexpression in U87-MG and U251 cells. **B** Western blot analysis of CA9 and ANXA1 protein. **C** ELISA of secreted ANXA1 in conditioned medium. **D** qRT-PCR of HIF-1α, CA9, and ANXA1 mRNA under normoxia versus hypoxia. **E** ELISA of secreted ANXA1 under normoxia versus hypoxia. **F** Dual-luciferase reporter assay with wild-type or HRE-mutant ANXA1 promoter. **G** Fold-change of ANXA1 mRNA induced by hypoxia in CA9-NC versus CA9-KD cells. **H** Hypoxia-induced ANXA1 secretion in CA9-NC versus CA9-KD cells. In vitro: ≥ 3 independent replicates. Western blots, representative images; β-actin, loading control. Data, mean ± SEM. Multi-group comparisons, one-way ANOVA with Tukey’s test. ***P < 0.001, ****P < 0.0001

To validate the proteomics findings, we assessed the effect of CA9 on ANXA1 transcription. qRT-PCR showed that CA9 knockdown reduced ANXA1 mRNA by approximately 62.3% in U87-MG (*P* < 0.001) and 57.8% in U251 (*P* < 0.001), while CA9 overexpression elevated ANXA1 mRNA by approximately 2.76-fold in U87-MG (*P* < 0.001) and 2.31-fold in U251 (*P* < 0.001) (Fig. 7A). Western blotting confirmed the same regulatory relationship at the protein level (Fig. 7B).

Because ANXA1 can serve as a secreted intercellular communicator, we also measured its extracellular abundance. ELISA demonstrated that CA9 knockdown markedly reduced secreted ANXA1: in U87-MG, concentrations fell from 512.8 ± 45.2 pg/mL to 186.4 ± 23.7 pg/mL (*P* < 0.001); similar reductions were observed in U251 (478.6 ± 41.3 pg/mL vs. 203.5 ± 28.9 pg/mL; *P* < 0.01) (Fig. 7C). These data establish that CA9 regulates ANXA1 both at the level of cellular expression and in terms of extracellular release.

### Hypoxia activates the CA9–ANXA1 axis, and CA9 is required for full ANXA1 induction

To elucidate the role of hypoxia in the CA9–ANXA1 axis, U87-MG cells and patient-derived GSCs were exposed to hypoxia (1% O₂, 48 h) or normoxia (21% O₂) as described in the Methods. Cells and conditioned medium were collected immediately at the end of the hypoxic period to minimize reoxygenation confounds. Hypoxia robustly upregulated HIF-1α, CA9, and ANXA1 mRNA in both models. In U87-MG, increases were approximately 3.2-fold (HIF-1α; *P* < 0.01), 8.47-fold (CA9; *P* < 0.001), and 3.18-fold (ANXA1; *P* < 0.01); in GSCs, the corresponding increases were approximately 2.8-fold (HIF-1α; *P* < 0.01), 11.32-fold (CA9; *P* < 0.001), and 4.15-fold (ANXA1; *P* < 0.001) (Fig. 7D). ELISA showed that hypoxia markedly enhanced ANXA1 secretion: from 245.6 ± 32.4 to 687.3 ± 52.8 pg/mL in U87-MG (*P* < 0.001) and from 312.4 ± 41.7 to 892.5 ± 67.3 pg/mL in GSCs (*P* < 0.001) (Fig. 7E).

To test whether HIF-1α can directly transactivate ANXA1, we employed wild-type and HRE-mutant ANXA1 promoter reporters. Hypoxia drove an approximately 3.52-fold increase in wild-type ANXA1 promoter reporter activity (P < 0.001), whereas mutation of the HRE core sequence (5’-ACGTG-3’ to 5’-AAAAG-3’) markedly attenuated this response (Fig. 7F). These data support HRE-dependent transcriptional activation of the ANXA1 promoter under hypoxic conditions, consistent with HIF-1α-responsive regulation.

Comparing CA9-NC and CA9-KD cells under hypoxia, we found that CA9 depletion substantially attenuated ANXA1 induction: the fold-increase dropped from approximately 3.24-fold (NC, U87-MG) to 1.52-fold (KD) and from approximately 2.87-fold (NC, U251) to 1.38-fold (KD) (Fig. 7G). ELISA corroborated this: CA9-KD cells secreted only 41.2% as much ANXA1 as CA9-NC cells under hypoxia (*P* < 0.001) (Fig. 7H). Although hypoxia/HIF-1α signaling can activate ANXA1 transcription through a functional HRE, CA9 is additionally required for the full hypoxic induction of ANXA1 expression and secretion, identifying CA9 as an amplifying node within this axis.

These results indicate that although HIF-1α can initiate ANXA1 transcription through a functional HRE, the full magnitude of hypoxia-driven ANXA1 expression and secretion is additionally dependent on CA9. CA9 should therefore be interpreted not as the sole upstream regulator of ANXA1, but as an amplifying node within the broader hypoxia-responsive program—one that links recurrence-associated MES-like GSC states to enhanced ANXA1-mediated paracrine output.

### SPP1⁺ immunosuppressive TAMs are significantly enriched in recurrent GBM

**Fig. 8:**
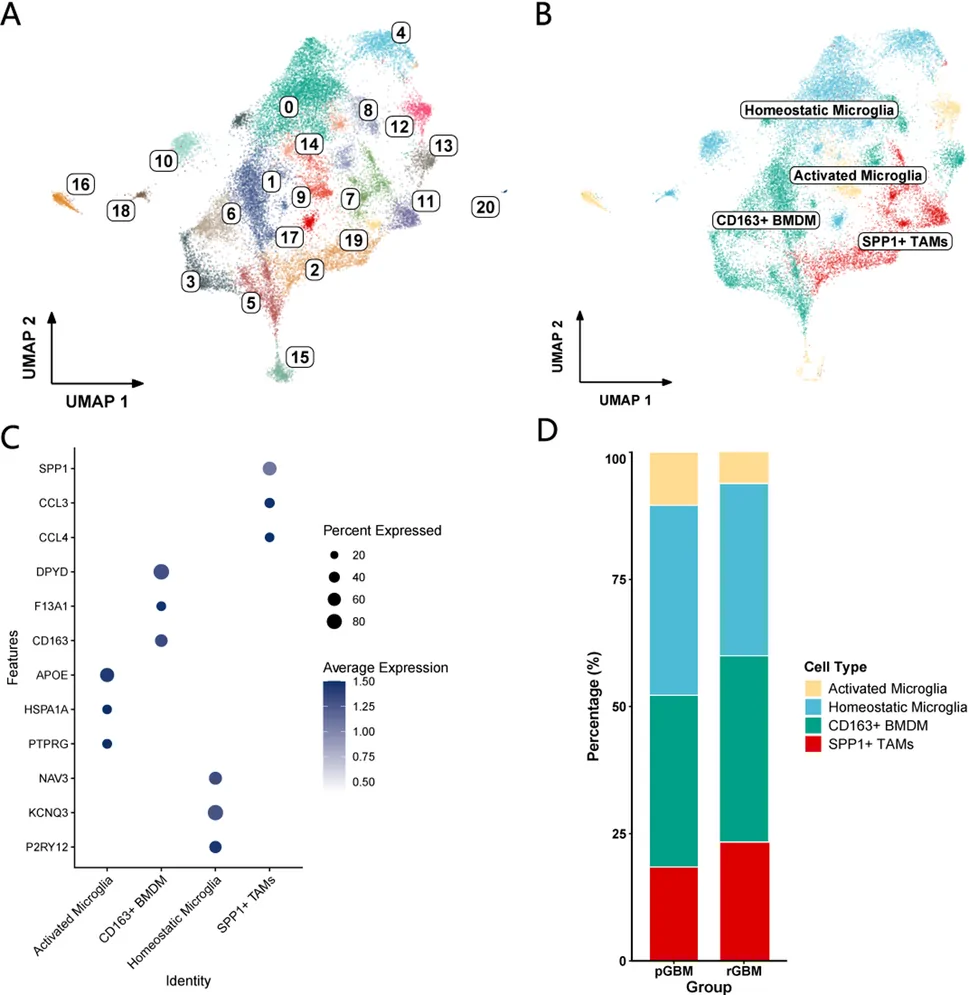
SPP1⁺ immunosuppressive TAMs are significantly enriched in recurrent GBM. **A** UMAP of re-clustered TAMs showing 21 clusters. **B** Annotation of four major TAM states: homeostatic microglia, activated microglia, CD163⁺ BMDMs, and SPP1⁺ TAMs. **C** Marker gene expression supporting TAM subtype annotations. **D** Proportional comparison of TAM subtypes between primary and recurrent GBM, showing SPP1⁺ TAM enrichment in recurrent samples

To characterize TAM heterogeneity within the GBM immune microenvironment, TAM populations were subjected to secondary clustering (resolution 0.5), yielding 21 clusters (Clusters 0–20; Fig. 8A) annotated into four major states: homeostatic microglia, activated microglia, CD163⁺ bone marrow-derived macrophages (BMDMs), and SPP1⁺ TAMs (Fig. 8B–C). SPP1⁺ TAMs were significantly more abundant in recurrent than in primary GBM, while activated microglia were relatively reduced (Fig. 8D). This compositional shift points to a progressive skewing of the macrophage compartment toward an immunosuppressive phenotype during disease recurrence.

### Cell-cell communication analysis identifies ANXA1–FPR1 as a leading candidate axis linking MES-like GSCs to SPP1⁺ TAMs

**Fig. 9:**
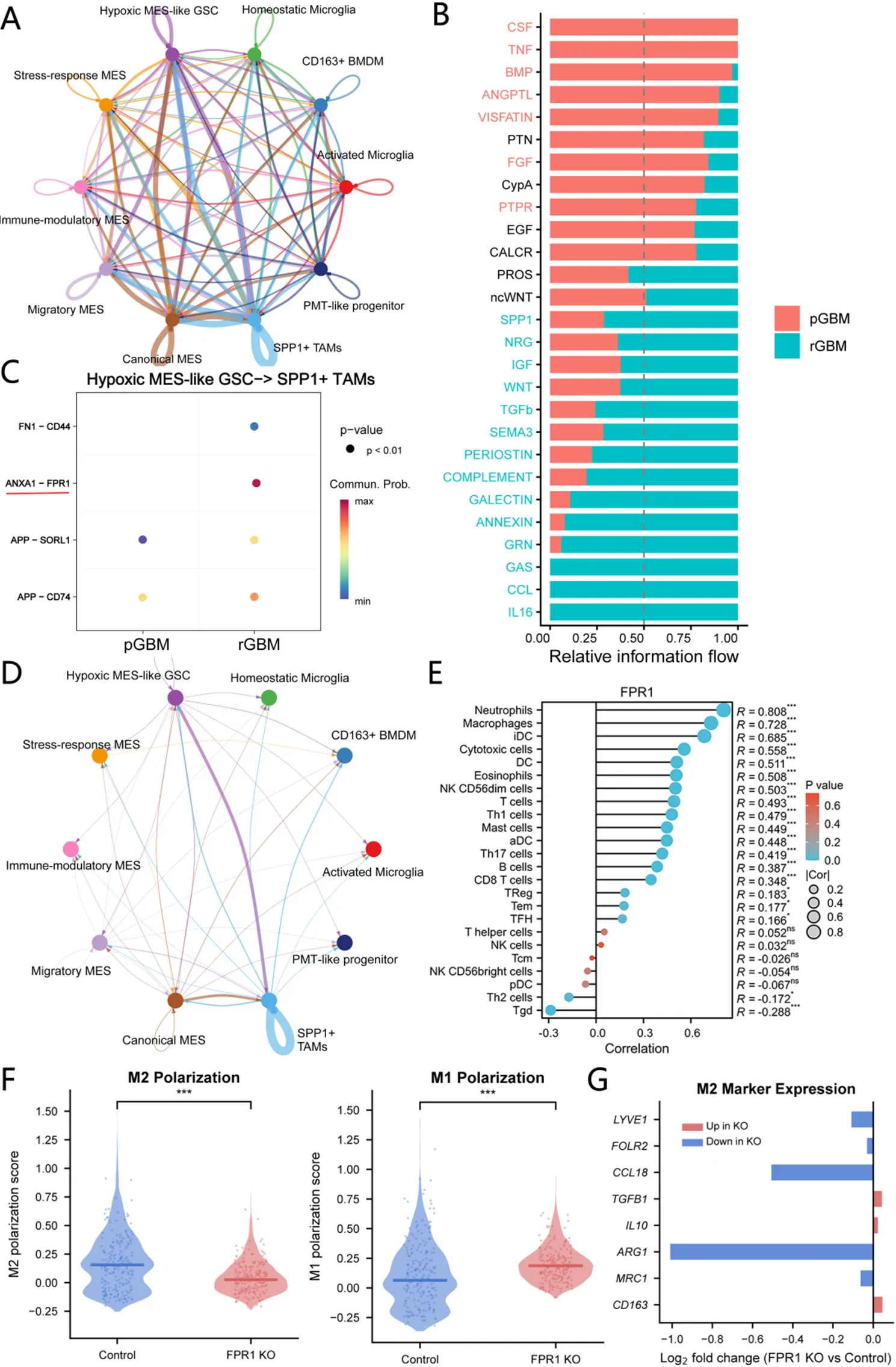
Cell-cell communication analysis identifies ANXA1–FPR1 as a leading candidate axis linking hypoxia-associated MES-like GSCs to SPP1⁺ TAMs. **A** CellChat analysis showing differential communication between hypoxia-associated MES-like GSCs and SPP1⁺ TAMs in primary versus recurrent GBM. **B** Pathway-level comparison of signaling activity. **C** Specific ligand–receptor pair analysis highlighting ANXA1–FPR1, FN1–CD44, and APP–CD74.**D** Cell-type-resolved visualization of ANXA1–FPR1 communication probability, highlighting the prominent involvement of the SPP1⁺ TAM-associated myeloid context. FPR1 is not interpreted as exclusively expressed by SPP1⁺ TAMs; this panel reflects communication probability inference rather than absolute receptor exclusivity. **E** Spearman correlations between ANXA1–FPR1 axis activity and immune cell abundance. **F** M1/M2 polarization score changes following in silico FPR1 knockdown in SPP1⁺ TAMs. **G** Expression changes of M2-associated genes. Color intensity or line thickness, communication probability or signal strength. *P < 0.05, **P < 0.01, ***P < 0.001

CellChat analysis revealed markedly enhanced intercellular communication between hypoxia-associated MES-like GSCs and SPP1⁺ TAMs in recurrent GBM (Fig. 9A). At the pathway level, SPP1, PROS, and NRG signaling were more active in recurrent samples (Fig. 9B). Among specific ligand–receptor pairs, ANXA1–FPR1, FN1–CD44, and APP–CD74 interactions were all strengthened in recurrent tumors, with ANXA1–FPR1 ranking as a top candidate axis (Fig. 9C). Among the recurrent GBM-enriched ligand–receptor interactions, ANXA1–FPR1 was prominently implicated in the communication context involving hypoxia-associated MES-like GSCs and SPP1⁺ TAMs (Fig. 9D). We note that FPR1 is not interpreted as an exclusive marker of SPP1⁺ TAMs; rather, the CellChat analysis indicates that FPR1-associated signaling contributes most prominently to the inferred intercellular communication involving this myeloid subpopulation in recurrent GBM. Notably, the nomination of ANXA1 as a candidate paracrine mediator based on proteomic and cohort analyses (Sect.  5) was independently supported by CellChat inference, which identified ANXA1–FPR1 as a leading ligand–receptor axis between hypoxia-associated MES-like GSCs and SPP1⁺ TAMs in recurrent GBM.This cross-method convergence further supported the prioritization of ANXA1 for functional validation.

ssGSEA analysis showed that ANXA1–FPR1 axis activity correlated positively with neutrophil (*R* = 0.808; *P* < 0.001) and macrophage (*R* = 0.728; *P* < 0.001) abundance (Fig. 9E). In silico FPR1 knockdown in SPP1⁺ TAMs led to a significant decline in M2 polarization scores alongside an increase in M1 scores (Fig. 9F) and downregulation of M2-associated genes including ARG1, CCL18, and LYVE1 (Fig. 9G). Although these results are computational in nature, they converge with our functional data to support the biological relevance of the ANXA1–FPR1 communication axis.

### Secreted ANXA1 mediates CA9-dependent M2-like macrophage polarization

**Fig. 10:**
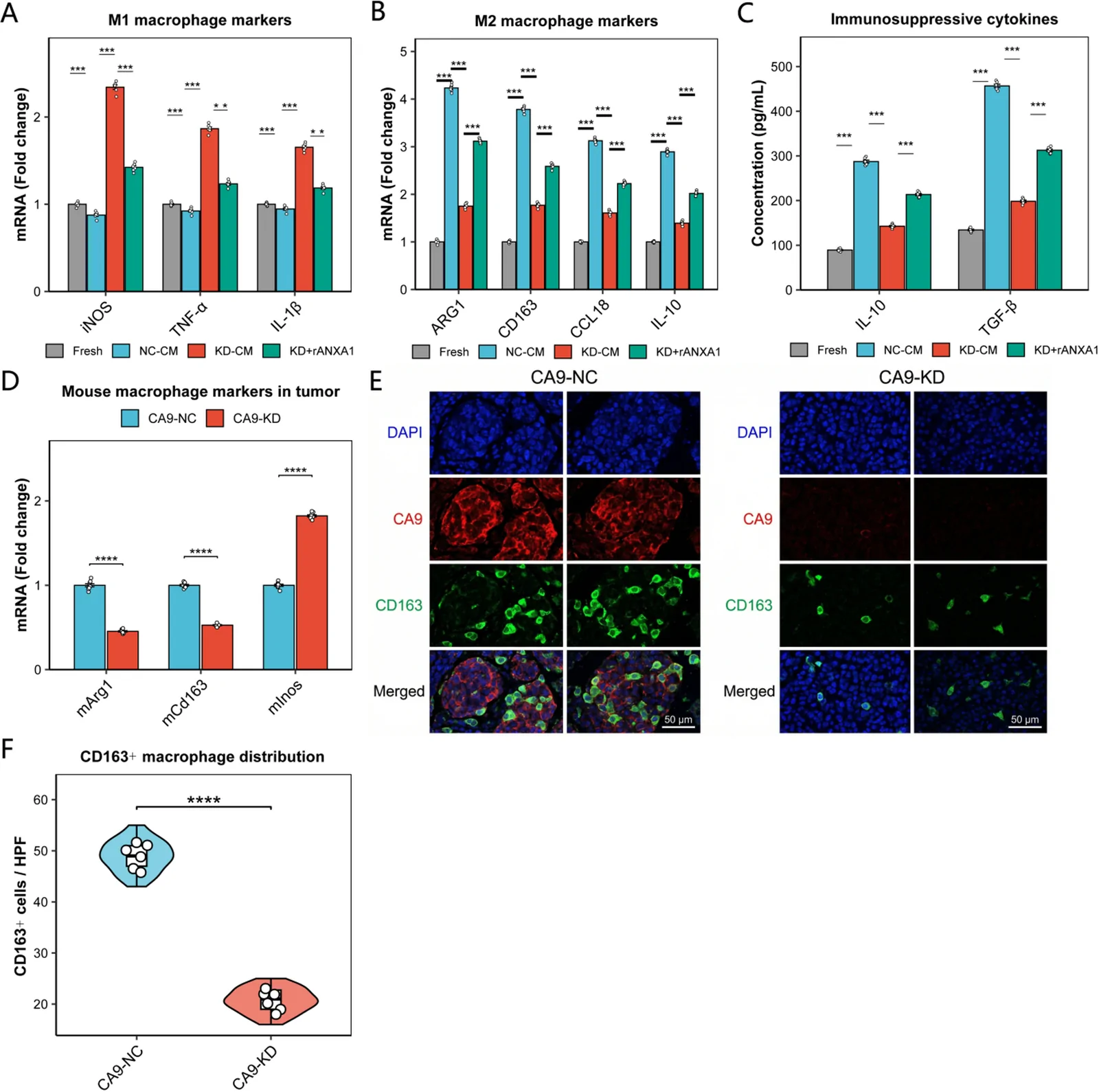
Secreted ANXA1 mediates CA9-dependent M2-like macrophage polarization and participates in immunosuppressive microenvironment formation in vivo. **A** qRT-PCR of M2-like markers (ARG1, CD163, CCL18, IL-10) in THP-1-derived macrophages under different conditions. **B** qRT-PCR of M1-like markers (iNOS, TNF-α, IL-1β). **C** ELISA of IL-10 and TGF-β in macrophage supernatants. Macrophages treated with fresh medium, CA9-NC CM, CA9-KD CM, or CA9-KD CM + rANXA1 (100 ng/mL) for 48 h. **D** Mouse-specific qRT-PCR of macrophage phenotype markers in xenograft tumor tissue. **E** Representative immunofluorescence co-staining for CA9 and CD163. **F** Quantification of CD163⁺ macrophage infiltration density. In vitro: ≥ 3 independent replicates. In vivo: *n* = 8 per group. Data, mean ± SEM. Multi-group comparisons, one-way ANOVA with Tukey’s test; two-group comparisons, two-sided Student’s t-test. **P < 0.01, ***P < 0.001

PMA-differentiated THP-1 macrophages were treated for 48 h with: (1) fresh medium; (2) CA9-NC CM; (3) CA9-KD CM; or (4) CA9-KD CM supplemented with rANXA1 (100 ng/mL). Relative to fresh medium, CA9-NC CM significantly upregulated M2-like markers ARG1, CD163, CCL18, and IL-10 (all *P* < 0.001; Fig. 10A), with no significant change in M1 markers iNOS, TNF-α, and IL-1β (Fig. 10B). CA9-KD CM substantially blunted M2-skewing: ARG1, CD163, CCL18, and IL-10 fell by approximately 58.7%, 53.2%, 48.5%, and 51.8% versus CA9-NC CM (all *P* < 0.01), while iNOS and TNF-α trended upward. Critically, supplementing CA9-KD CM with rANXA1 significantly rescued M2-like marker expression, restoring ARG1 to approximately 73.6%, CD163 to approximately 68.4%, CCL18 to approximately 71.2%, and IL-10 to approximately 69.8% of CA9-NC CM levels (all *P* < 0.001). ELISA further confirmed that CA9-NC CM enhanced macrophage secretion of IL-10 and TGF-β, which was reduced by CA9-KD CM and partially restored by rANXA1 supplementation (Fig. 10C). This loss-of-function/rescue evidence chain supports secreted ANXA1 as a contributory paracrine mediator of CA9-dependent macrophage M2-like polarization. Because recombinant ANXA1 was used here as a rescue component supplemented to CA9-KD conditioned medium rather than as a standalone treatment, whether ANXA1 alone is sufficient to fully recapitulate the polarizing effect of CA9-high conditioned medium remains to be determined in future studies incorporating rANXA1-alone and dose-response experimental designs.

### CA9 knockdown remodels the immunosuppressive microenvironment in vivo

To evaluate the in vivo impact of CA9 on the TME, we interrogated xenograft tumor tissue from the nude mouse model. Mouse-specific qRT-PCR revealed that CA9-KD tumors expressed significantly lower levels of the murine M2-like markers mArg1 and mCd163 and higher levels of the M1-like marker mInos relative to CA9-NC tumors (Fig. 10D). Immunofluorescence co-staining confirmed that CD163⁺ macrophage infiltration density was substantially reduced in CA9-KD tumors: 21.4 ± 4.2 cells/HPF versus 48.7 ± 6.3 cells/HPF in CA9-NC tumors (*P* < 0.01) (Fig. 10E–F). These in vivo data indicate that CA9 loss suppresses tumor growth and is accompanied by a phenotypic shift away from M2-like macrophage infiltration, supporting a role for CA9 in shaping the immunosuppressive TME.

### FPR1 mediates the CA9-ANXA1 axis-driven macrophage M2-like polarization

**Fig. 11:**
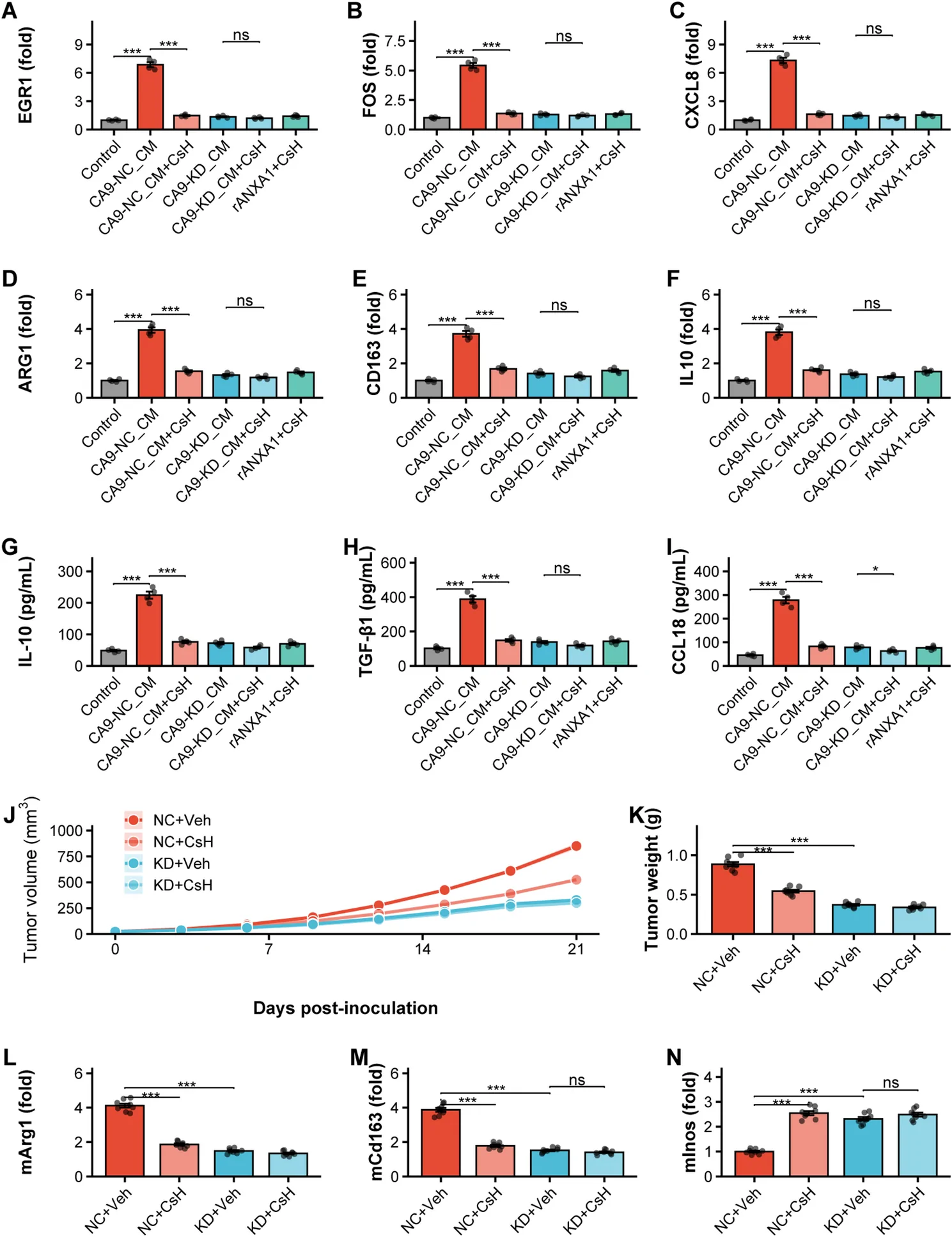
FPR1 mediates the CA9-ANXA1 axis-driven macrophage M2-like polarization. **A**-**C** qRT-PCR analysis of FPR1-responsive immediate-early genes (EGR1, FOS, CXCL8) in THP-1-derived macrophages following 1 h stimulation with conditioned medium (CM) from CA9-NC or CA9-KD glioma cells, with or without FPR1 antagonist cyclosporin H (CsH, 10 micromolar). **D**-**F** qRT-PCR analysis of M2-like polarization markers (ARG1, CD163, IL10) after 48 h stimulation. **G**-**I** ELISA quantification of secreted cytokines (IL-10, TGF-beta1, CCL18) in macrophage supernatants after 48 h treatment. **J** Tumor growth curves. Mice bearing CA9-NC or CA9-KD U87-MG xenografts were treated with vehicle or CsH (10 mg/kg, intraperitoneally, every other day) from day 3 (*n* = 8 per group). **K** Endpoint tumor weights. **L**-**N** Mouse-specific qRT-PCR analysis of macrophage markers (Arg1, Cd163, Nos2) in tumor tissues. Data are mean +/- SEM. *P < 0.05, **P < 0.01, ***P < 0.001; ns, not significant

To establish that secreted ANXA1 acts through FPR1 to mediate macrophage polarization, we treated THP-1-derived macrophages with conditioned medium (CM) from CA9-NC or CA9-KD cells in the presence or absence of the selective FPR1 antagonist cyclosporin H (CsH, 10 µM). We first assessed activation of FPR1-responsive immediate-early genes after short-term (1 h) stimulation. CA9-NC CM robustly induced EGR1, FOS, and CXCL8 expression compared with control (all *P* < 0.001; Fig. 11A-C), whereas CsH pretreatment almost completely abolished this induction and reduced expression to levels comparable with CA9-KD CM (all *P* < 0.001 vs. CA9-NC CM; Fig. 11A-C).

Consistent with impaired FPR1 signaling, CsH treatment substantially attenuated M2-like polarization. After 48 h stimulation, expression of ARG1, CD163, and IL10 was markedly reduced to levels approximating those observed with CA9-KD CM treatment (all *P* < 0.001; Fig. 11D-F). ELISA analysis further corroborated these findings: CA9-NC CM induced robust secretion of IL-10, TGF-β1, and CCL18, whereas CsH pretreatment dramatically reduced these levels (all *P* < 0.001; Fig. 11G-I). Importantly, CsH showed no substantial additional inhibitory effect on CA9-KD CM, providing epistatic support that FPR1 acts downstream of the CA9-ANXA1 axis.

To validate these findings in vivo, we administered CsH to nude mice bearing CA9-NC or CA9-KD xenografts. CsH treatment significantly suppressed tumor growth in the CA9-NC group (*P* < 0.001; Fig. 11J) and reduced tumor weight (*P* < 0.001; Fig. 11K), whereas it produced no meaningful additional antitumor effect in mice bearing CA9-KD tumors (*P* > 0.05; Fig. 11J-K). Mouse-specific qRT-PCR analysis demonstrated that CsH reduced M2-like markers (mArg1, mCd163) and increased the M1-like marker mInos in CA9-NC tumors (all *P* < 0.001; Fig. 11L-N), while producing no significant changes in CA9-KD tumors. Taken together, these pharmacological and epistatic experiments support FPR1 as a functionally relevant receptor mediating ANXA1-driven macrophage polarization downstream of CA9.

## Discussion

Despite meaningful advances in multimodality treatment, GBM prognosis remains dismal, and recurrence is virtually inevitable [2–8]. Recurrence is far more than simple tumor regrowth; it reflects the convergence of cellular state reprogramming, therapy-imposed selection, and deep microenvironmental remodeling [6, 7, 14, 38]. GSCs occupy a central position in this process, bridging treatment resistance, sustained proliferation, and recurrence initiation [9–13]. Understanding what drives GSC malignancy and their crosstalk with the immune microenvironment in rGBM therefore carries important therapeutic implications.

Prior work has linked CA9 to hypoxic adaptation, enhanced invasiveness, and poor outcome across multiple malignancies [29–36]. We confirm broad CA9 upregulation in GBM, with further elevation in the mesenchymal subtype and in recurrent disease, each correlating with shorter survival. Functional experiments demonstrate that these associations are not merely correlative: CA9 knockdown suppresses proliferation, induces apoptosis, restores temozolomide sensitivity, and inhibits tumor growth in vivo. CA9 is therefore an active contributor to malignant GBM phenotypes, not merely a passive hypoxia marker.

Recognition of GSC heterogeneity has deepened considerably [11, 14, 15]. Our single-cell analysis adds an immunologically relevant dimension by showing that CA9 is selectively enriched in a hypoxia-associated MES-like GSC subpopulation that expands in recurrent tumors, harbors elevated stemness, and is characterized by activated DNA repair, HIF-1, and Wnt signaling programs. These observations provide single-cell resolution evidence for state remodeling during recurrence and help explain how hypoxic stem-like tumor states may interface with myeloid remodeling [10, 13, 20].

The canonical function of CA9 is to regulate intra- and extracellular acid-base balance, enabling survival and invasion in hostile hypoxic-acidic environments [30, 33, 34]. In GBM, this capacity likely has multiple downstream consequences: intracellular pH regulation may sustain proliferative signaling, while extracellular acidification can amplify immunosuppression, facilitate matrix remodeling, and reinforce the GSC niche [26, 28, 35, 36]. The temozolomide sensitivity data reported here further suggest that CA9 contributes to GSC-associated drug resistance, although the complete mechanistic picture remains to be resolved.

Beyond cell-intrinsic effects, a central finding is that CA9 appears to shape the tumor microenvironment through paracrine signaling. Quantitative proteomics identified ANXA1 as a prominent downstream candidate, and molecular validation confirmed that CA9 positively regulates ANXA1 at both transcriptional and secretory levels. ANXA1 participates in tumor invasion, migration, inflammation, and immune evasion across several cancer types [21–24, 46]. Our data show that CA9 knockdown reduces not only intracellular ANXA1 abundance but also its extracellular release, positioning CA9 as a regulator of a secreted immunomodulatory signal with the capacity to influence the surrounding myeloid environment. It should be noted that ANXA1 represents a prioritized and functionally validated downstream mediator identified through multi-layered screening—encompassing proteomic differential expression, biological relevance to immune regulation and secretory signaling, public cohort correlations, and macrophage abundance associations—rather than the only possible effector of CA9. Other CA9-regulated proteins identified in our proteomic dataset may similarly contribute to the recurrent GBM microenvironment and warrant future investigation.

Importantly, our data do not argue that ANXA1 is uniquely controlled by CA9 or independent of canonical HIF-dependent signaling. Previous studies have reported hypoxia-responsive regulation of ANXA1 [47], and our promoter-reporter data are consistent with HRE-dependent transcriptional activation under hypoxia. The additional contribution of the present study is to demonstrate that CA9 depletion attenuates the full amplitude of hypoxia-driven ANXA1 induction and secretion—an effect that cannot be fully explained by reduced HIF-1α activity alone, given that HIF-1α levels were similarly elevated under hypoxia in both CA9-NC and CA9-KD cells. We therefore propose that CA9 acts as a recurrence-associated, hypoxia-responsive amplifier linking hypoxic MES-like GSC states to ANXA1-mediated myeloid remodeling, rather than as the sole determinant of ANXA1 expression.

TAMs are central architects of the immunosuppressive GBM microenvironment, and GSCs can reprogram them toward pro-tumorigenic phenotypes through multiple signaling axes [17–20, 48, 49]. Our TAM subpopulation analysis uncovered marked enrichment of SPP1 + TAMs alongside relative loss of activated microglia in recurrent GBM, indicating that recurrence involves fundamental restructuring of the myeloid compartment. CellChat inference identified enhanced ligand-receptor communication between hypoxia-associated MES-like GSCs and SPP1 + TAMs in recurrent tumors, with ANXA1-FPR1 emerging as a leading candidate axis. In silico FPR1 perturbation in SPP1 + TAMs reduced M2 polarization scores and M2-associated gene expression, providing computational support that is consistent with the functional experiments. FPR1 should not be considered an exclusive marker of SPP1⁺ TAMs. Other myeloid subsets within the GBM microenvironment may also express FPR1 and potentially respond to tumor-derived ANXA1 signals. Our conclusion is restricted to the observation that, within the CellChat communication inference framework, FPR1-associated signaling was most prominently implicated in the SPP1⁺ TAM-related communication context in recurrent GBM. Future studies incorporating FPR1 protein-level mapping across TAM subsets will be needed to fully resolve its cellular distribution.

ANXA1–FPR1 signaling has previously been implicated in inflammatory regulation and macrophage polarization in various biological contexts. Therefore, the novelty of the present study does not lie in identifying ANXA1–FPR1 as a macrophage-regulatory pathway per se. Rather, the principal contribution is to embed this pathway within a recurrent GBM-specific neuroimmune context: we identify CA9-high hypoxia-associated MES-like GSCs as a recurrence-enriched tumor-cell state that amplifies ANXA1 expression and secretion, thereby coupling hypoxic stem-like tumor programs to FPR1-associated immunosuppressive myeloid remodeling. The upstream tumor-cell state identity, CA9 as an amplifying node, and the connection to SPP1⁺ TAM enrichment in recurrent GBM represent the primary mechanistic additions to existing knowledge.

The conditioned-medium and ANXA1 rescue experiments provide the direct experimental anchor for the proposed paracrine model. CA9-high GBM cells promoted M2-like macrophage polarization, this effect was substantially diminished by CA9 knockdown, and exogenous recombinant ANXA1 partially but significantly restored polarization capacity. Pharmacological FPR1 blockade with cyclosporin H further attenuated immediate-early transcriptional responses and M2-like polarization markers induced by CA9-NC conditioned medium. Integrating single-cell, proteomic, and functional data, we propose a recurrence-associated neuroimmune crosstalk model. In this model, CA9-high hypoxia-associated MES-like GSCs are not merely passive hypoxic responders; they act as paracrine sources that enhance ANXA1 expression and secretion. Secreted ANXA1 engages FPR1-associated signaling in TAMs—particularly within the SPP1⁺ TAM-associated communication context—thereby contributing to immunosuppressive myeloid remodeling in recurrent GBM [21–24, 45, 46]. The novelty of this model lies in identifying a recurrent GBM-specific upstream cellular state and a CA9-dependent amplification mechanism that links hypoxic stem-like tumor programs to neuroimmune remodeling, rather than in redefining ANXA1–FPR1 as a macrophage-regulatory pathway.

In vivo data reinforce these conclusions. Although the subcutaneous nude mouse model does not fully replicate the intracranial GBM niche or a complete immune system, CA9-knockdown tumors nonetheless showed reduced murine M2-like macrophage marker expression and fewer CD163-positive macrophage infiltrates. In the CA9-intact setting, cyclosporin H treatment also reduced tumor growth and M2-like macrophage-associated signals, further supporting a role for FPR1-associated signaling downstream of tumor-derived ANXA1.

Several limitations merit acknowledgment. The paired clinical cohort is small, and the immunofluorescence findings require confirmation in larger multicenter series. The in vivo experiments used a subcutaneous xenograft model in nude mice, which does not fully recapitulate the intracranial GBM neuroimmune niche and lacks a complete T-cell compartment. Therefore, these findings should be interpreted as supportive evidence for macrophage-associated effects of the CA9–ANXA1–FPR1 axis rather than as a comprehensive reconstruction of the human recurrent GBM immune microenvironment. THP-1-derived macrophages, while widely used, do not perfectly recapitulate primary macrophage biology. Although pharmacological FPR1 blockade with cyclosporin H supports involvement of FPR1 downstream of the CA9-ANXA1 axis, receptor-specific knockdown or knockout experiments in macrophages would further strengthen mechanistic causality. Although our dual-luciferase reporter assay supports a functional contribution of the identified HRE motif to hypoxia-responsive ANXA1 promoter activation, it does not directly demonstrate endogenous HIF-1α chromatin occupancy at this locus. Future chromatin immunoprecipitation (ChIP-qPCR) or CUT&RUN experiments will be important to confirm direct HIF-1α binding at the endogenous ANXA1 promoter. In addition, the recombinant ANXA1 experiment was designed as a rescue assay to evaluate whether ANXA1 contributes to the macrophage-polarizing activity of CA9-high conditioned medium, rather than as a sufficiency assay. Because an rANXA1-alone treatment group was not included, we cannot fully distinguish the direct macrophage-polarizing effect of ANXA1 alone from its context-dependent activity within the broader tumor-cell secretome. Finally, the precise mechanism by which CA9 amplifies hypoxia-driven ANXA1 expression and secretion remains unresolved.

## Conclusions

Integrating data from clinical specimens, public cohorts, single-cell transcriptomics, quantitative proteomics, and in vitro and in vivo functional experiments, this study demonstrates that CA9 is enriched in hypoxia-associated MES-like GSCs in recurrent GBM and participates in paracrine crosstalk with macrophages by promoting ANXA1 expression and secretion. The resulting ANXA1-FPR1-associated signaling contributes to immunosuppressive TAM polarization and supports a recurrent GBM neuroimmune niche. These findings identify CA9 as a recurrence-associated molecular node that links hypoxic MES-like GSC states to ANXA1–FPR1-associated immunosuppressive myeloid remodeling in recurrent GBM, providing a framework for understanding neuroimmune crosstalk at the intersection of hypoxic adaptation and myeloid reprogramming.

## Supplementary Information

Supplementary Material 1.

Supplementary Material 2.

## Data Availability

The public datasets used in this study are available in the following repositories: GEO under accession number GSE174554, TCGA through the UCSC Xena platform (https://xenabrowser.net/), and the CGGA mRNAseq_693 cohort through the CGGA portal (http://www.cgga.org.cn/). The data generated during the current study are available from the corresponding authors on reasonable request.
