# Supplementary material for: Hypoxic CA9-high mesenchymal-like glioma stem cells promote ANXA1-FPR1-dependent macrophage polarization in recurrent glioblastoma
Source: J Neuroinflammation. 2026 Jun 29;23:315. doi: 10.1186/s12974-026-03905-0 (PMC13579895; doi:10.1186/s12974-026-03905-0)
Supplement: Supplementary file 1 [file 12974_2026_3905_MOESM2_ESM.doc]

Figure S1. CA9 knockdown suppresses CA9 mRNA expression and inhibits tumor growth in a subcutaneous xenograft model.(A) Quantitative real-time PCR (qRT-PCR) analysis demonstrating significant downregulation of CA9 mRNA expression in U251 and U87-MG cells following lentivirus-mediated CA9 knockdown (CA9-KD) compared with negative control (CA9-NC) cells. Data are normalized to β-actin and presented as mean ± SEM from three independent experiments. Statistical significance was determined by two-tailed Student's t-test. ***P < 0.001.

(B) Terminal tumor weights from BALB/c nude mice bearing subcutaneous U87-MG xenografts. Mice were inoculated with CA9-NC or CA9-KD cells (5 × 10⁶ cells per mouse) and sacrificed at day 25 post-implantation. Tumors were excised and weighed. Data are presented as mean ± SEM (n = 8 per group). Statistical significance was determined by two-tailed Student's t-test.***P < 0.001.

Figure S2. Clustering results of tumor cells.(A) After normalization, tumor cells were clustered into 30 subclusters (Clusters 0–29).(B) After normalization, MES-like cells were clustered into 13 subclusters (Clusters 0–12).
